# Early life exposure of infants to benzylpenicillin and gentamicin is associated with a persistent amplification of the gut resistome

**DOI:** 10.1186/s40168-023-01732-6

**Published:** 2024-02-03

**Authors:** Dhrati V. Patangia, Ghjuvan Grimaud, Carol-Anne O’Shea, C. A. Ryan, Eugene Dempsey, Catherine Stanton, R. Paul Ross

**Affiliations:** 1 School of Microbiology, University College Cork, Cork, Ireland; 2 Teagasc Food Research Centre, Moorepark, Fermoy Co., Cork, Ireland; 3 APC Microbiome Ireland, Cork, Ireland; 4 Department of Paediatrics and Child Health, University College Cork, Cork, Ireland; 5 Infant Research Centre, University College Cork, Cork, Ireland

**Keywords:** Infant gut, Gut microbiota, Shotgun metagenomics, Resistome profile, Strain persistence

## Abstract

**Background:**

Infant gut microbiota is highly malleable, but the long-term longitudinal impact of antibiotic exposure in early life, together with the mode of delivery on infant gut microbiota and resistome, is not extensively studied.

**Methods:**

Two hundred and eight samples from 45 infants collected from birth until 2 years of age over five time points (week 1, 4, 8, 24, year 2) were analysed. Based on shotgun metagenomics, the gut microbial composition and resistome profile were compared in the early life of infants divided into three groups: vaginal delivery/no-antibiotic in the first 4 days of life, C-section/no-antibiotic in the first 4 days of life, and C-section/antibiotic exposed in first 4 days of life. Gentamycin and benzylpenicillin were the most commonly administered antibiotics during this cohort’s first week of life.

**Results:**

Newborn gut microbial composition differed in all three groups, with higher diversity and stable composition seen at 2 years of age, compared to week 1. An increase in microbial diversity from week 1 to week 4 only in the C-section/antibiotic-exposed group reflects the effect of antibiotic use in the first 4 days of life, with a gradual increase thereafter. Overall, a relative abundance of Actinobacteria and *Bacteroides* was significantly higher in vaginal delivery/no-antibiotic while Proteobacteria was higher in C-section/antibiotic-exposed infants. Strains from species belonging to *Bifidobacterium* and Bacteroidetes were generally persistent colonisers, with *Bifidobacterium breve* and *Bifidobacterium bifidum* species being the major persistent colonisers in all three groups. *Bacteroides* persistence was dominant in the vaginal delivery/no-antibiotic group, with species * Bacteroides ovatus* and *Phocaeicola vulgatus* found to be persistent colonisers in the no-antibiotic groups. Most strains carrying antibiotic-resistance genes belonged to phyla Proteobacteria and Firmicutes, with the C-section/antibiotic-exposed group presenting a higher frequency of antibiotic-resistance genes (ARGs).

**Conclusion:**

These data show that antibiotic exposure has an immediate and persistent effect on the gut microbiome in early life. As such, the two antibiotics used in the study selected for strains (mainly Proteobacteria) which were multiple drug-resistant (MDR), presumably a reflection of their evolutionary lineage of historical exposures—leading to what can be an extensive and diverse resistome.

Video Abstract

**Supplementary Information:**

The online version contains supplementary material available at 10.1186/s40168-023-01732-6.

## Background

Early life is a crucial window for the development of the neonate as the foundations for later life are laid. The timing and source of colonisation of the microbiota play an indispensable role in developing optimal health. These initial colonisers perform important functions such as the breakdown of complex compounds, e.g., the role of bifidobacteria in the breakdown of human milk oligosaccharides (HMOs) to harvest energy and synthesise vitamins and metabolites such as vitamins B and K and short-chain fatty acids (SCFAs) [1]. They protect against pathogen colonisation—a phenomenon referred to as colonisation resistance [2], and they are involved in neural development and immune programming as the neurocognitive and immunity maturation occur during this period [3–5].

Infants have a simple gut microbial structure comprising fewer bacterial species than adults, stabilising by 3 to 4 years of age towards adult life. This early-life gut microbiota is influenced by several factors that include but are not limited to gestational age [6], mode of delivery (vaginal vs C-section) [7], feeding habits (breastfeeding vs formula feeding) [8], and antibiotic uptake, both by the infant and the mother [9–12].

The infant gut is considered an antibiotic resistance gene (ARG) reservoir [13, 14]. This high ARG abundance is associated with Gammaproteobacteria, owing to their intrinsic resistance and high colonisation levels in early life [15–20]. C-section (CS) born infants are colonised by a high abundance of opportunistic pathogens belonging to taxa such as *Escherichia coli*, *Klebsiella*, and *Enterobacter*, often resulting in higher antibiotic use and ARG carriage in these infants [21, 22]. Antibiotic resistance is a global health concern [23]. Septic infections caused by antibiotic-resistant bacteria are responsible for 214,000 neonatal deaths each year, of which multi-drug resistant (MDR) bacteria cause 30% [24]. The immediate effect after antibiotic treatment is generally an overall decrease in gut microbiota diversity, including commensals, benefiting the colonisation of antimicrobial-resistant (AMR) strains due to selective pressure [25]. Increasing evidence has demonstrated the association between antibiotic use and altered microbiota in early life, the rise in ARGs [11, 26], and its linkage to the development of disease conditions in later life [27].

CS and antibiotic use are common medical practices [28, 29], but both disturb the natural microbial colonisation process, impacting the long-term health of the host [30, 31]. However, there are still important gaps in the understanding of how disruption of microbiota by differing modes of delivery and antibiotic use can enhance or reduce the gut resistome as the infant ages. In this study, the combined effects of CS birth mode and antibiotic uptake on the meta-taxonomic diversity, and frequency of ARGs over the first few weeks of life were investigated by comparing it to CS-born and vaginally delivered control groups without antibiotics. Furthermore, the effect of antibiotic exposure in early life on the resistome and functional profile of infant gut microbiota longitudinally up to 2 years of age was investigated. CS delivery and antibiotic exposure in early life were seen to pose a twofold disadvantage, resulting in significantly lower microbial diversity carrying a higher ARG frequency with antibiotic use being the dominant influencing factor. Furthermore, the impact of antibiotic use in early life resonated up to 2 years of age with amplified resistome profile observed in antibiotic-exposed infants.

## Methods

### Study group participants

In this study, DNA extracted from faecal samples from full-term infants from two study cohorts—INFANTMET and MYNEWGUT—were used to investigate the evolution of the infant gut resistome using shotgun metagenomics throughout the first 24 weeks of life up to 2 years of age. MYNEWGUT (MNG) recruited full-term CS-born infants administered antibiotics in the first 4 days of life. INFANTMET (IM) included infants born at full term via CS and vaginal delivery and were not administered antibiotics in the first week of life [32]. A subset of subjects from these two studies (*n* = 45) were selected randomly and divided into three groups. Group 1 (MNG + ab/CSab) (*n* = 10) included infants born by CS and given antibiotics during the first week of life. Group 2 (IM-Ab/CSnoab) (*n* = 17) contains infants born by CS, not administered antibiotics, and group 3 (IM-Ab/VDnoab) (*n* = 18) has infants born via vaginal delivery (VD) not administered antibiotics in the first few days of life. The cohorts and control groups were matched by gestational age (> 35 weeks of gestation), birth mode (CS and VD), antibiotic treatment (i.e. benzylpenicillin and gentamicin), diet (mostly breastfed or combination of breast and formula feeding), and time points of sample collection. The demographics for these three infant groups are as follows (Table 1).

**Table 1:** Clinical metadata for infants included in this study

**Summary statistics (*n* = 45)**				
**Characteristics**	**MNG-CS (CSab)**	**IM-CS (CSnoab)**	**IM-VD (VDnoab)**	
**Number of infants, *n* (%)**	10 (22.22)	17 (37.77)	18 (40)	
**Number of samples, *n* (%)**	50 (24)	78 (37.5)	80 (38.4)	
**Time points, *n***				
Week 1	10 (%)	17	18	
Week 4	10	15	17	
Week 8	10	17	15	
Week 24	10	17	17	
Year 2	10	12	14	
**Gender, *n* (%)**				
Male	4 (40)	9 (52.9)	9 (50)	
Female	6 (60)	8 (47)	9 (50)	
**Mode of delivery, *n* (%)**				
Vaginal	0	0	18 (100)	
C-section	10 (100)	17 (100)	0	
**Gestational age (days)**	*280 (273,287)	*273 (272,28)	278 (277,287)	1 missing
**Birth weight (g)**	4002 +—646.61	3543.5 + -463.88	3499.4 + -402.9	
**Antibiotic uptake in the first 4 days of life**	Yes	No	No	
**Feeding, *n* (%)**				
Breast	1 (1)	3 (17.6)	4 (25)	
Combine	8 (8)	10 (58.8)	14 (77.7)	
NA	1 (1)	4 (23.5)	0 (0)	
**Maternal age at infant birth (years) – average**	NA	34	33	
**Gravidity**	1.5(1,2)	2(2,4)	2(1.25,2.75)	
**Parity**	1(1,1)	2(2,3)	2(1,2.75)	
**APGAR score**				
Initial	9(8.25,9)	9(9,9)	9(8.75,9)	
Second	10(9,10)	10(9,10)	10(9,10)	

### DNA extraction

Metagenomic DNA was extracted from fecal samples using the QIAmp Fast DNA Stool Mini kit (Qiagen, UK) with a modified protocol combined with a repeated bead beating method [33]. Briefly, 1 ml of lysis buffer was added to the stool sample in the bead-beating tube. The samples were homogenised with the lysis buffer using a mini beadbeater, incubated at 70 °C for 15 min with mixing at regular intervals, followed by another 15-min incubation at 37 °C, and then centrifuged at 4 °C for 5 min at 16,000 × g. The supernatant was then treated with ammonium acetate to remove impurities and incubated on ice for 5 min. This was followed by another centrifugation step at 16,000 × g at 4 °C for 10 min, and then, the DNA was precipitated by combining with an equal volume of isopropanol and stored overnight at − 20 °C. The next day, the DNA was pelleted by centrifugation at 16,000 × g at 4 °C for 15 min, washed with ethanol and Tris–EDTA, and treated with Proteinase K, RNAse, and purified according to the manufacturer’s instructions (QIAmp Fast DNA Stool Mini kit; Qiagen, UK). The DNA was quantified using Qubit and stored at − 30 °C until further use.

### Shotgun sequencing library preparation

The concentration of DNA was determined using Qubit and diluted to 0.2 ng/µl as per the manufacturer’s instructions. Library prep for shotgun sequencing was performed using a Nextera XT kit following the Nextera XT DNA Library Preparation Guide from Illumina. Briefly, genomic DNA (0.2 ng/µl) was tagmented in 20 µl PCR reaction (containing 5 µl of gDNA, 10 µl of tagment DNA buffer, and 5 µl of Amplicon Tagment Mix) volume at 55 °C. The tagmented DNA was then amplified using Illumina index primers, following which the libraries were cleaned using AMPure beads. The quality of the library was then evaluated by running it on the Bioanalyzer using the Agilent High Sensitivity DNA chip. The DNA was quantified using Qubit, normalised, pooled, and outsourced to the Teagasc sequencing facility for sequencing. Samples were sequenced as part of three sequencing runs along with one single sample (I159) ran on a new run making it part of a fourth sequencing run.

### Filtering, taxonomic, and functional analysis of metagenomics reads

The quality of raw reads from metagenomic sequencing was assessed using FastQC (v0.11.8) and MultiQC (v1.9). Further, trimming, filtering, and host genome decontamination of the metagenomics reads were done using Trimmomatic (v0.39) and bowtie2 with *Homo sapiens* reference genome using the default parameters via the KneadData (v0.7.2) program from Huttenhower lab (https://github.com/biobakery/kneaddata). Further HUMAnN3 (v3.0) was used for the functional assignment of the reads, providing microbial gene and pathway abundance information [34], while MetaPhlAn3 was used to provide the taxonomic assignment to the filtered and trimmed reads using default parameters. MetaPhlAn3 output was processed using the microbiomics (v0.0.0.9000) and phyloseq (v1.38.0) package in R (v4.2.1), and relative abundance and mean relative abundance data were used for plotting figures in R. To check for possible batch-effects based on our grouping and sequencing plate variable, we used the R package MBECS v1.4.0 with ComBat as the batch effect correcting algorithm [35]. Briefly, ComBat uses a non-parametric empirical Bayes framework to correct for batch effects. Then, we determined beta-diversity using the Bray–Curtis distance and a PCoA with the corrected batch-effect data. We further calculated the PERMANOVA for all our study variables and compared them with the PERMANOVA based on uncorrected data. We obtained *p* values and *R*^2^ within a 0.0001% range of variation, so we considered that batch effects are negligible.

### Metagenomics assembly and contig binning

Post-KneadData filtering, the quality-filtered sequencing reads were first fixed and sorted using bbmap (v38.22). The metagenomes were then assembled using metaSPAdes (v3.14) [36] with default parameters. The scaffolds generated were filtered to obtain resulting scaffolds with a minimum length of 1000 bp (1kbp). Metagenomic bins were generated from the filtered scaffolds using three binning tools (MetaBAT v2.12.1, MaxBin v2.2.622, and CONCOCT v1.0.023) using metaWRAP (v1.3.1) [37] with default parameters. The bins were then refined with Bin_refinement module of metaWRAP with options ‘-c 50—× 10,’ corresponding to the criterion of medium-quality draft metagenome assembled genomes (MAGs). The quality (estimated completeness and contamination) of bins was evaluated with CheckM (v1.0.18) [38], implemented in metaWRAP. This resulted in the formation of 1449 medium-quality MAGs which were used for further analysis.

### Taxonomic and functional annotation of MAGs

The resulting MAGs were then provided taxonomic annotation with GTDB-Tk (v1.5.0) using ‘classify_wf’ workflow with default settings [39]. The phylogenetic tree for the 1449 MAGs was generated using PhyloPhlAn (v3.0.2) with options ‘-diversity low’ and ‘-fast’ [40]. The protein-coding genes from MAGs were predicted using Prokka (v1.14) using default parameters. dbCAN (v3.0.1) [41] (run_dbcan.py) was used to assign carbohydrate-active enzymes (CAZymes) to all the MAGs using default parameters. Only certain CAZymes as reported to be involved in HMO and FOS metabolism were filtered and examined in downstream analysis. inStrain [42] was used for the identification of identical strains from MAGs, briefly, MAGs were dereplicated using drep (v3.2.0) resulting in 598 unique MAGs which were then mapped on filtered and trimmed shotgun reads using the inStrain profile command using Bowtie2 (v2.3.4). The inStrain profiles are then processed with inStrain compare, where the profiles are compared and those with 99.99% population level—average nucleotide identity (popANI) are termed to be the same strain, belonging to the same cluster and thus considered belonging to the same sample. The resulting strains were then classified as early or late colonisers, if they appeared during the first 8 weeks (early colonisers, *t* ≤ 8 weeks) of life or later (late colonisers, *t* > 8 weeks). Early colonisers were further classified as persistent colonisers and non-persistent colonisers. The definition for persistent colonisers was adapted from Lou et al. [43] and persistent colonisers were defined as early colonisers that persisted for 24 weeks (6 months) or more (t ≥ 24 weeks); otherwise, the strain was termed as non-persistent coloniser (*t* < 24 weeks).

To look for possible contaminants, we examined strains (i.e. same strain with popANI > 99.99%) detected in the same inStrain cluster (set of genomes having popANI > 99.99%) coming from different individuals (supplementary Table S1). If the samples where these strains were detected as identical came from different sequencing plates, we did not classify them as contaminants. These could be individuals living in the same household or siblings (e.g. I08 and I18). Only three samples (M15, M20, M25) from the same sequencing plate and corresponding to different (*n* = 3) individuals were detected as containing the same strains (*n* = 2). These strains were classified as possible contaminants and removed from the analysis. We also looked at possible contaminants using the blanks included in the sequencing runs. No contamination was found using this method.

### Resistome and virulence analysis

MAGs generated were then processed through ABRicate (https://github.com/tseemann/abricate) using default settings with CARD (Comprehensive Antibiotic Resistance Database) [44], VFDB (Virulence Factor Database) [45], and plasmid finder [46] databases. Results from CARD and VFDB were used further unless otherwise specified. RGI-bwt and CARD (v3.1.4) [47] were used to predict ARGs in the clean and filtered metagenomics reads using default parameters. The RGI-bwt read mapping results at the gene level were normalised by the read counts per sample, converted to copies per million (cpm) of ARGs per sample, and used for downstream analysis. ARG names from ABRicate and RGI output were curated to antibiotic class names based on phenotype from CARD. Any gene that conferred resistance to three or more antibiotic classes was termed MDR in downstream analysis. However, when the antibiotic classes of interest in the study (classes of antibiotics administered to infants in the first week of life in the CSab group—Aminoglycoside and or Penams) appeared, we spelled them out along with MDR for better visualisation purposes.

### Differential abundance analysis

Analysis of which taxa or ARG classes drive the difference between the three groups in the present study was done using MaAsLin2 [48] and Songbird [49]. The MaAsLin2 model was fit with overall data, and at week 1 and week 4, treating the group as a fixed effect with each of the three study groups (VDnoab, CSnoab, CSab) used as a reference with min_prevalance = 0.1 and min_abundance = 0. For differential abundance analysis of longitudinal data, Songbird was performed using default parameters to study the bacterial species most associated with each group and driving the differences in the groups (Formula: C(Group, Treatment('VDnoab')) and C(Group, Treatment('CSnoab'))). Only the top 10 associations (both positive and negative top 10) were used for further analysis.

### *Statistical analysis and data representation*

**Fig. 1:**
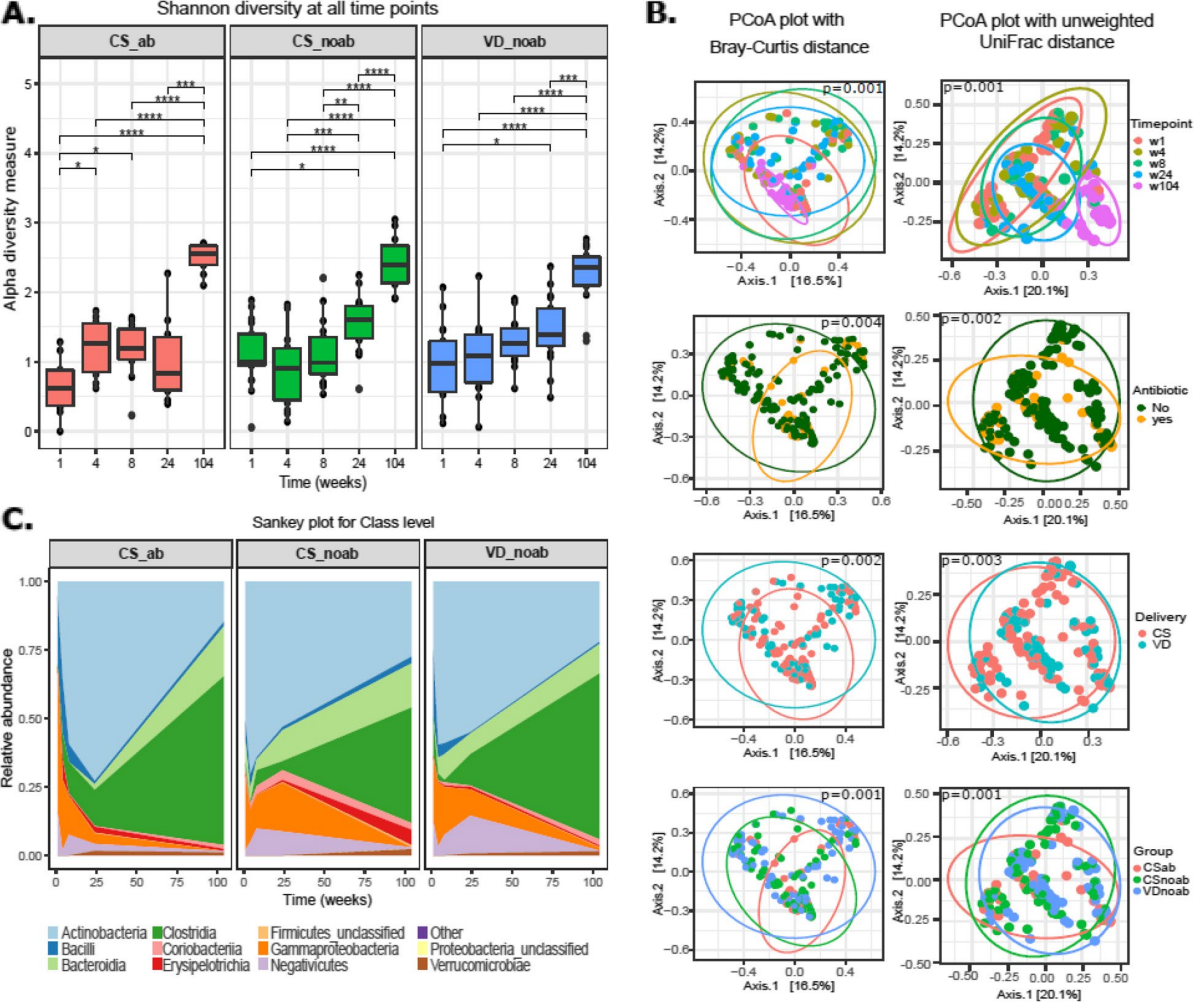
**A** Alpha-diversity as measured by Shannon’s diversity index between all time points in the three study groups. *P* values were calculated using the Wilcoxon test, with p.adjusted values < 0.05 used as the significance threshold. **B** PCoA plot using Bray–Curtis and unweighted UniFrac distance metrics; with ellipses drawn with each grouping variable namely time-point, antibiotic, delivery, and study groups (confidence interval: 90%). *P* value and *R*^2^ values were obtained from PERMANOVA using pairwiseAdonis. **C** Taxonomic distribution of infant gut microbiota at class level using relative abundance for each group over time from week 1 to year 2

Downstream analysis of the data was done in R (v4.1.0) using packages including Vegan (v2.5.7) [50], Phyloseq (v1.38.0) [51], ggplot2 (v3.3.5) [52], microbiome utilities (v1.0.16), and ggpubR (v0.4.0). Statistical analysis was performed in R using Permanova to check the proportion of explained variance and significance of each variable using pairwiseAdonis (v0.4). Wilcoxon sum tests, paired *t* tests, Dunn test, and ANOVA as mentioned throughout the text were used to verify statistical significance. Spearman correlation coefficient was used to determine the correlation between relative abundance over time for species for each group, and the top 10 positive and negative results were plotted in R. Statistical significance was determined by 999 permutations, and *p* values were corrected using FDR; *p* values below 0.05 were considered significant. To assess the power of the study, we carried out a post hoc statistical power analysis. We used the ‘simr’ package (v1.0.7) and ‘lme4’ package (v1.1) in R, which determines the power for linear mixed effects models based on Monte Carlo simulations. Briefly, we first fitted the model using the function ‘lmer’ on the second axis of the PCoA with Bray–Curtis distance (Fig. 1B), with the following parameters: ‘Axis.2 ~ Group*time + (1|id)’, where ‘Axis.2’ is the second axis of the PCoA, ‘Group’ is the grouping variable in our study, and ‘id’ is the identity of the infant (i.e., the random variable). Then, we created a fitted lmer model using the function ‘makeLmer’ with the above formula and used the output of lmer. Finally, we assessed statistical power for delivery mode using the ‘powerSim’ function as follows: ‘powerSim(model, nsim = 1000, test = fcompare(Axis.2 ~ time))’, where ‘model’ is the fitter lmer model. We obtained a statistical power of 96.10% with alpha = 0.05 and 1000 simulations.

## Results

To characterise the microbiome and resistome development in early life, metagenomic data of two hundred and eight stool samples from 45 infants were divided into three groups based on their mode of delivery and antibiotic exposure in the first week of life, i.e., vaginal delivery/no-antibiotic (VDnoab); C-Section/no-antibiotic (CSnoab); C-Section/antibiotic (CSab). This study included samples at five time points from week 1, week 4, week 8, week 24, and year 2 of age. Altogether 10 infants were born by CS and administered antibiotics in the first 4 days of life, with gentamycin and benzylpenicillin being the most commonly administered. Gestational age and birth weight were similar across all groups (Table 1).

### Antibiotic use affects microbial composition and decreases diversity

Metagenomics analysis revealed increased richness and alpha-diversity (Shannon index) over time in all groups (Fig. 1A, 1B), with significant differences between all early time points (week 1, week 4, week 8, and week 24) and the final time-point, year 2. The significant difference (p.adj-value = 0.029 Wilcoxon test with BH) in alpha-diversity (Shannon index) observed between week 1 and week 4 in the CSab group, which was not observed in the other groups, shows an increase in microbial diversity in very early life possibly due to the effect of antibiotic administration (Fig. 1A). The CSab group had low initial microbial diversity (Shannon index) when compared with the other groups (Figure S1A), with a significant difference observed at week 1 between CSab and CSnoab (p.adj-value = 0.03, Wilcoxon test with BH) groups and at week 24 between CSab and CSnoab (p.adj-value = 0.039, Wilcoxon test with BH); however, no significant differences were observed otherwise (Figure S1A).

We then calculated beta-diversity using Bray–Curtis dissimilarity and Unifrac distance and visualised it using principal coordinate analysis (PCoA) to look at differences between microbial profiles (Fig. 1B). Using Bray–Curtis distances, the CSab group showed a distinct microbial composition compared to CSnoab (*p* value = 0.0105, PERMANOVA) and VDnoab (*p* value = 0.0060, PERMANOVA) groups. Beta-diversity also varied between time points (*p* value = 0.001, R2 = 0.11; Adonis2), by mode of delivery (*p* value = 0.003, *R*^2^ = 0.013; Adonis2), by duration of breastfeeding (*p* value = 0.001, *R*^2^ = 0.06), and antibiotic use (*p* value = 0.002, *R*^2^ = 0.016; Adonis2) in early life; with antibiotic use explaining slightly higher variation between the groups after the time and feeding variables. Microbial communities were mainly structured by time-point, and significant differences were observed between the CSab and VDnoab groups at week 1, week 4, and week 8 (*p* values = 0.024, 0.048, 0.018, respectively, Adonis2) and between VDnoab and CSnoab groups at week 1 (*p* value = 0.075) (Figure S1B). To determine the effect of delivery mode and antibiotic use on the microbial composition, PERMANOVA was performed using pairwiseAdonis in R on microbial composition data at each time-point. At week 1, delivery mode and antibiotic usage caused significant variations in the microbial composition of infants, with delivery mode explaining higher variance (pairwiseAdonis). The effect of the mode of delivery decreased over time, but antibiotic use in the first week of life was a significant variable affecting microbial composition in infants up to week 24 (Figure S2B).

### Microbial composition of infants in early life evolves with age

Given the significant effect of delivery mode and early antibiotic exposure on microbial diversity, the colonisation pattern in the three groups was examined. The phyla Firmicutes and Proteobacteria were prevalent in CS-born infants, with the highest relative abundance in the CSab group (Figure S2A). Actinobacteria abundance was relatively high in VD infants, with high relative abundance in the no-antibiotic-treated groups and lowest in the CSab group from week 1 (At week 1: p.adj value: VDnoab to CSab = 0.0005, Dunn test with BH). A detailed look at each time point demonstrated the absence of phylum Bacteroidota in the CS-born infants at week 1 with higher relative abundance in the no-antibiotic groups (p.adj-value: VDnoab to CSnoab = 0.003, VDnoab to CSab = 0.008, Dunn test with BH). At weeks 1 and 4, Firmicutes were relatively higher in the CS-born infants (CSnoab) (Figure S2A) (at week 1: p.adj-value: CSnoab to VDnoab = 0.009, at week 4: p.adj-value: CSab to CSnoab = 0.005 and CSab to VDnoab = 0.002, Dunn test with BH).

**Fig. 2:**
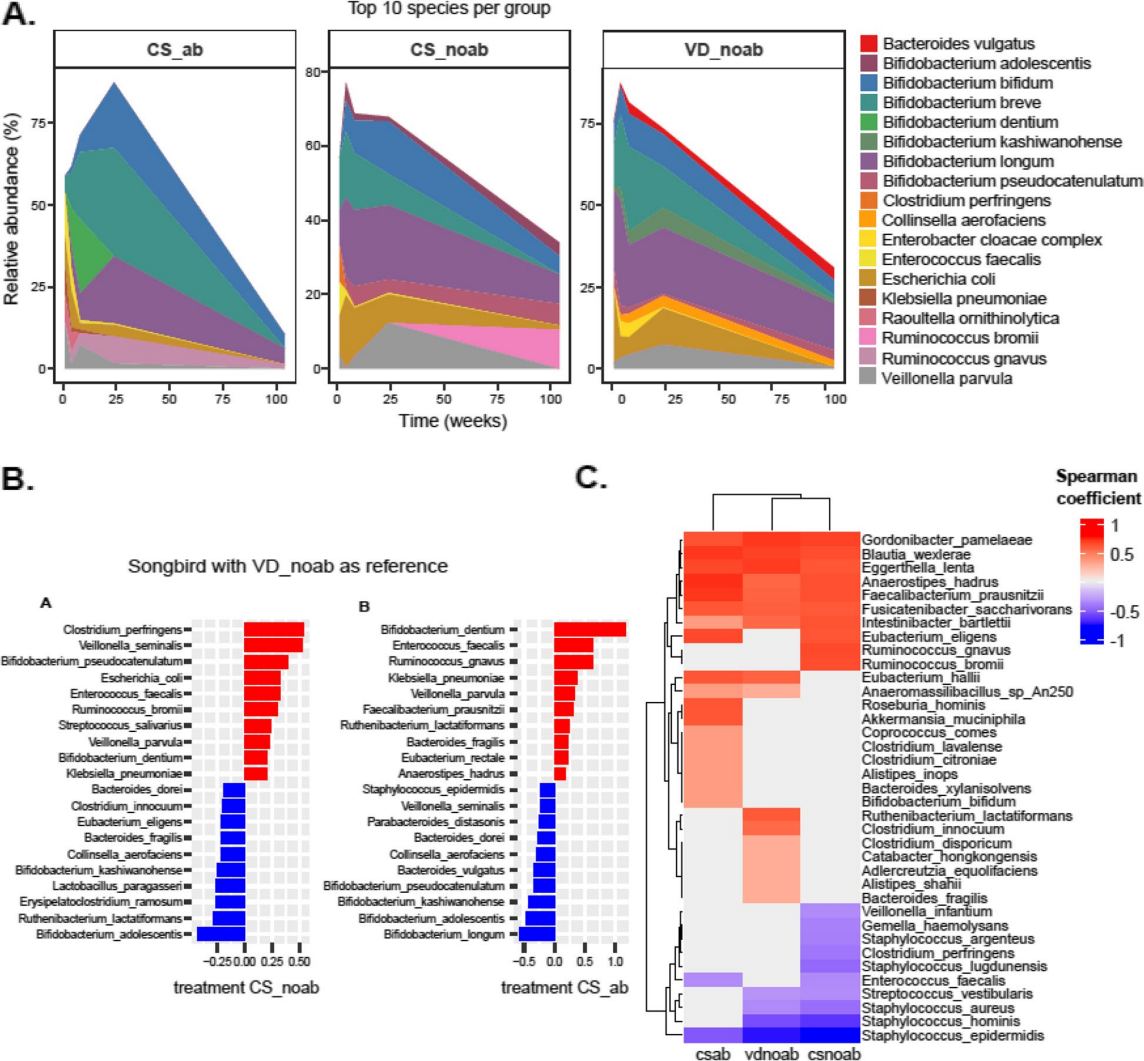
**A** Relative abundance (%) showing the top 10 species per group over time from week 1 up to year 2, where time is in weeks. **B** Plots show differential abundance analysis using Songbird with VDnoab group as reference run using the formula C(Group, Treatment('VDnoab')). The plot on the left side corresponds to the CSnoab group as treatment while that on the right depicts the CSab group as treatment against the reference group. In both cases, negative values (blue bars) represent the association to the reference group (here VDnoab) while positive values (red bars) represent the association to the treatment group; here CSnoab (on left plot) and CSab (right side plot). **C** Heatmap representing the association of bacterial abundance with time. Results from Spearman correlation analysis using the relative abundance of species for each group to time-point. *P* values were adjusted using BH, those < 0.05 were considered significant, and corresponding species and their coefficients were plotted. In the plot, blue bands denote decreasing with time (negative coefficient), red bands denote positive coefficient which means increasing over time, while grey means NA denoting not detected significantly as top 10 in that group

We further examined the mean relative abundances of the classes (Fig. 1C) and the top 10 most abundant species for all groups longitudinally (Fig. 2A). Commensals such as *Bifidobacterium* species including *B. bifidum*, *B. breve*, *B. longum*, *B. kashiwanohense*, *Bacteroides/Phocaeicola* (*Phocaeicola vulgatus*), *Collinsella aerofaciens*, and *E. coli* abundances were highest in the VDnoab group at week 1 and week 4, and generally, an increasing trend was seen for all groups until week 24. Interestingly, *Bifidobacterium dentium* abundance was highest in the CSab group, granting this species an undisclosed benefit in adverse circumstances like the administration of antibiotics. A low prevalence of commensals was observed in CS-born infants, with higher initial relative abundance of opportunistic bacteria in the CSab group belonging to Proteobacteria and Firmicutes such as *Klebsiella pneumoniae*, *Ruminococcus gnavus*, *Veillonella parvula*, *Raoultella ornithinolytica*, and *Enterobacter cloacae* complex, which decreased over time (Fig. 2A, C). The prevalence of the top 10 species occurring over time in the three groups is provided in Table 2.

**Table 2:** Prevalence of the top 10 species per group over time

VDnoab	Prevalence
s__*Bifidobacterium_longum*	0.219614
s__*Bifidobacterium_breve*	0.151354
s__*Escherichia_coli*	0.090879
s__*Bifidobacterium_bifidum*	0.078704
s__*Veillonella_parvula*	0.034615
s__*Bifidobacterium_kashiwanohense*	0.032284
s__*Bifidobacterium_pseudocatenulatum*	0.028567
s__*Phocaeicola_vulgatus*	0.023586
s__*Collinsella_aerofaciens*	0.022992
s__*Enterobacter_cloacae_complex*	0.018023
**CSnoab**	
s__*Bifidobacterium_longum*	0.157771
s__*Bifidobacterium_breve*	0.111949
s__*Escherichia_coli*	0.104861
s__*Bifidobacterium_bifidum*	0.072274
s__*Veillonella_parvula*	0.038586
s__*Bifidobacterium_pseudocatenulatum*	0.036361
s__*Bifidobacterium_adolescentis*	0.022993
s__*Clostridium_perfringens*	0.021653
s__*Enterococcus_faecalis*	0.021367
s__*Ruminococcus_bromii*	0.021031
**CSab**	
s__*Bifidobacterium_breve*	0.138188
s__*Bifidobacterium_longum*	0.081597
s__*Bifidobacterium_dentium*	0.069409
s__*Bifidobacterium_bifidum*	0.062468
s__*Escherichia_coli*	0.050663
s__*Veillonella_parvula*	0.048931
s__*Enterococcus_faecalis*	0.040728
s__*Ruminococcus_gnavus*	0.033023
s__*Raoultella_ornithinolytica*	0.029764
s__*Klebsiella_pneumoniae*	0.024008

### Stronger association and colonisation of CS-born babies with pathobionts

To further determine the association of each group and time with bacterial taxa, microbial relative abundance (species) data were associated with longitudinal time-point using Spearman correlation for each group (Fig. 2C). The top 10 positive and negative coefficients derived after filtering for significant *p*.adjusted values were then plotted (Wilcoxon test and BH method). In general, facultative anaerobes (such as *Staphylococcus*, *Streptococcus*, *Enterococcus*) known to colonise the gut in the first week of life showed a decreasing trend over time, while obligate anaerobes (*Blautia*, *Bifidobacterium*, *Anaerostipes*, *Bacteroides*, *Eggerthella*, *Ruminococcus*, *Eubacterium*) showed a growing trend with time [53]. The taxa *B. bifidum*, *Faecalibacterium prausnitzii*, *Blautia* (*Blautia wexlerae*), *Bacteroides* (*Bacteroides fragilis*), *Eubacterium* (*Eubacterium rectale*, *Eubacterium hallii*), *Anaerostipes* (*Anaerostipes hadrus*), and *Ruminococcus* (*Ruminococcus bromii*) were found to have positive associations and increased in all three groups over time (Fig. 2C).

Species that were significantly different between any two groups overall were identified using the Songbird differential abundance analysis tool. Briefly, Songbird results showed a stronger association of *Bifidobacterium* species to the VDnoab group, but when CSnoab to CSab were compared, *Bifidobacterium* species were more associated with the CSnoab group (Table S2). When the VDnoab group was used as a reference (Fig. 2B), several commensals with high relative abundance and prevalence (Fig. 2A and Table 2) were associated with it, some of which are involved in folate, lactate, and butyrate production such as *Bifidobacterium adolescentis*, *Ruthenibacterium lactatiformans*, *Erysipelatoclostridium ramosum*, *B. longum*, *Lactobacillus paragasseri*, *B. kashiwanohense*, *C. aerofaciens*, *B. fragilis*, *Parabacteroides distasonis*, and *Phocaeicola dorei*. Conversely, a mix of opportunistic bacteria and commensals including *Clostridium perfringens*, *Veillonella* (sp *seminalis* and *parvula*), *Bifidobacterium pseudocatenulatum*, *Streptococcus salivarius*, *E. coli*, *Enterococcus faecalis*, and *R. bromii* was associated with the CSnoab group. Similarly, the CSab group was more associated with *B. dentium*, *Ruminococcus gnavus*, *E. faecalis*, *V. parvula*, *Faecalibacterium prausnitzii*, and *K. pneumoniae*.

When comparing associations between CSnoab vs CSab groups (Figure S2C), we observed similar results as above where *B. dentium*, *V. parvula*, *K. pneumoniae*, *F. prausnitzii*, *B. breve*, *E. faecalis*, and *R. gnavus* showed a stronger association to the CSab group (Figure S2C).

### Antibiotic administration in early life is strongly associated with the resistome profile

**Fig. 3:**
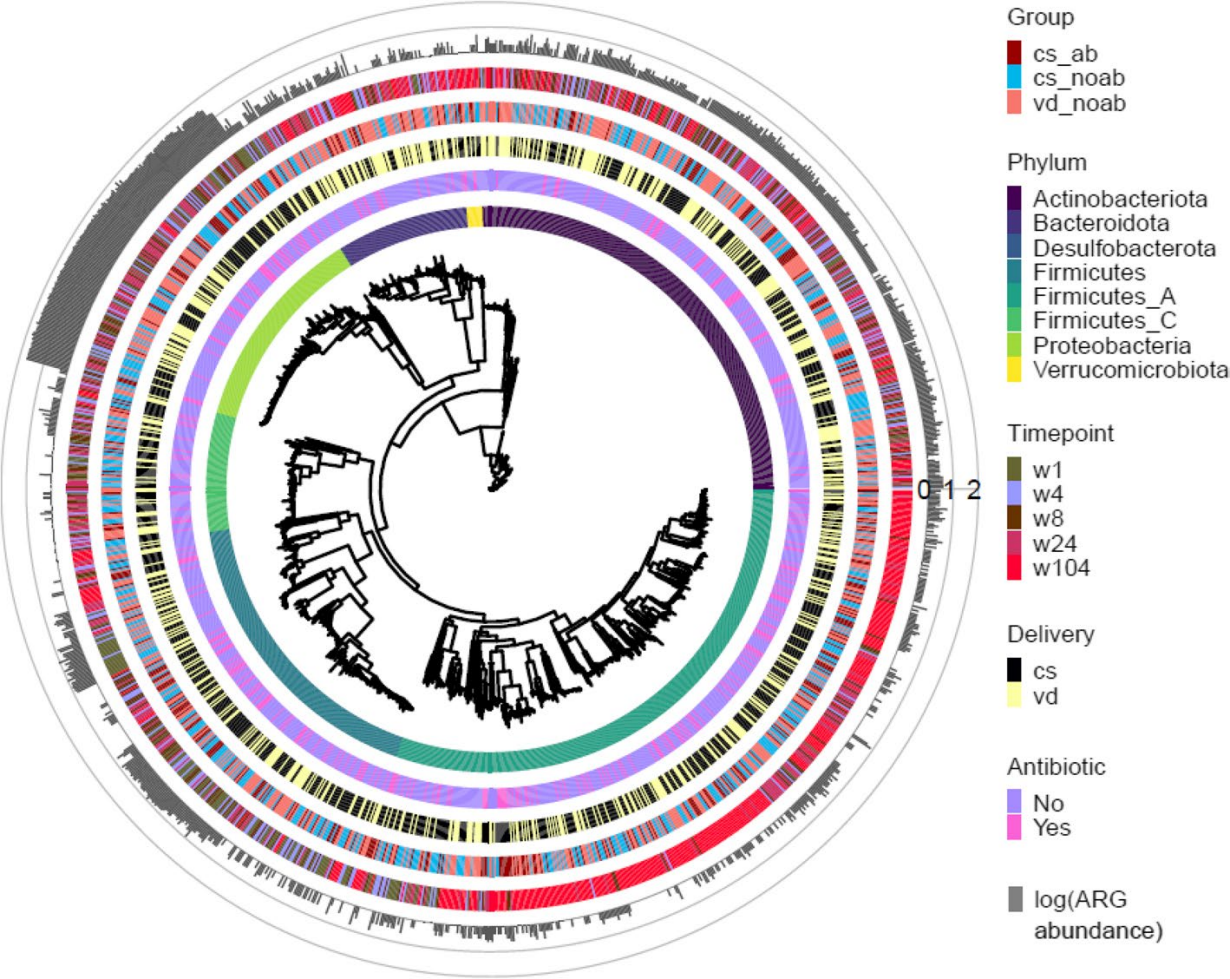
Phylogenetic tree created using Phylophlan from 1448 medium-quality MAGs generated in this study. Each branch represents a MAG with (from innermost to outermost ring): phylum level distribution of the MAGs, antibiotic use in early life and delivery mode, groups variable in study, succeeded by time-point and log-transformed genome counts of ARG abundance per MAG as examined using ABRicate with CARD

To further analyse the data from a genome perspective, we reconstructed a total of 1448 medium-quality prokaryotic MAGs corresponding to eight phyla, 1441 known species (GTDB-k), and 49 unknown species. Using universal markers, we reconstructed a phylogenetic tree using PhyloPhlan (Fig. 3).

**Fig. 4:**
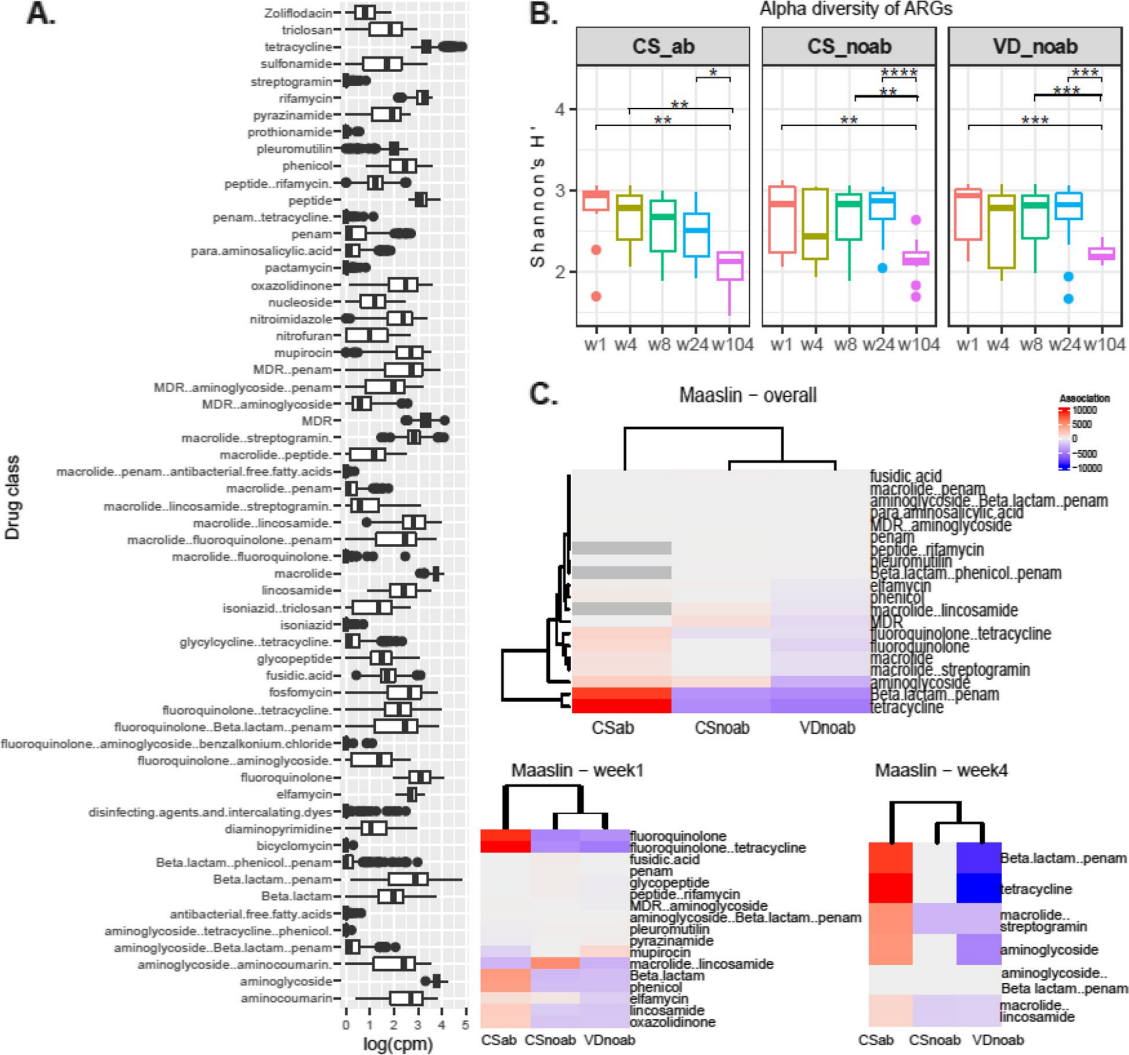
**A** Box plot representing the total resistome profile of the infants included in this study, with antibiotic class names of penams and aminoglycosides retained in MDR classes to facilitate understanding, as these are the classes of antibiotics administered to infants in the CSab group. Antibiotic classes were detected using RGI with CARD. The abundance of antibiotic classes was normalised to cpm and log-transformed. **B** Alpha-diversity plot using Shannon diversity index for ARG diversity for each group between all the time points. *P* value of < 0.05 was considered significant (Wilcoxon test). **C** MaAsLin2 differential abundance analysis coefficients obtained by running MaAsLin2 overall and at week 1 and week 4 using Group as a fixed effect with each group (VDnoab, CSnoab, and CSab) as reference per run along with the parameters min_prevalence = 0.1 and min_abundance = 0

We annotated the MAGs using the CARD database with ABRicate. The presence of 213 ARGs was detected in 1024 MAGs. Resistance was observed against 26 different classes of antibiotics in the infant cohort, which included antimicrobials belonging to ‘critically important’, ‘highly important’, and ‘important’ categories (as categorised by WHO: https://apps.who.int/iris/bitstream/handle/10665/312266/9789241515528-eng.pdf). The highest ARG distribution was in the phylum Proteobacteria followed by Actinobacteria and Firmicutes (Fig. 3), with Proteobacteria possessing the highest abundance of unique ARGs (182). The majority of genera contributing to this ARG abundance included several taxa belonging to Gammaproteobacteria namely *Escherichia*, *Klebsiella*, *Enterobacter*, *Raoultella*, *Citrobacter*, and others including *Enterococcus*, *Staphylococcus*, *Streptococcus*, and *Bifidobacterium*. Interestingly, species belonging to *Escherichia* contributed to the largest number of ARG abundance accounting for 56% of the total number of ARGs detected (146 different ARGs present out of 213 detected) (Figure S3 A). Results from the read-based approach using RGI with CARD further confirmed the resistome profile distribution in infants with a high abundance of resistance genes observed for aminoglycoside, MDR, macrolide, beta-lactam, tetracycline, and fluoroquinolone antibiotics (Fig. 4A). Functional characterisation of ARGs showed that antibiotic target alteration was the most common type, followed by antibiotic inactivation and antibiotic efflux as mechanisms for antibiotic resistance. The distribution and abundance pattern of ARGs in the study’s three groups was investigated, and a decrease in alpha-diversity from week 1 to year 2 (p.adjusted values using Wilcoxon test with BH: for CSab = 0.01, for CSnoab = 0.012, and for VDnoab = 0.0005), week 4 to year 2 for CSab group only (p.adjusted value = 0.01, Wilcoxon test with BH), week 8 to year 2 (p.adjusted values using Wilcoxon test with BH: for CSnoab = 0.01 and for VDnoab = 0.00031) and week 24 to year 2 (p.adjusted values: for CSab = 0.03, for CSnoab = 0.00026, and for VDnoab = 0.00052, Wilcoxon test with BH) was observed; however, no other differences were seen (Fig. 4B). PERMANOVA demonstrated discrete clustering between the CSab and VDnoab groups (*p* value = 0.03, PERMANOVA) using a non-metric multidimensional scaling (NMDS) plot with Bray–Curtis distance matrix (Figure S3 B). Antibiotic use in early life and time both had a significant impact on the ARG diversity (antibiotic exposure: *p* value = 0.007, time: *p* value = 0.001, PERMANOVA) but no significant effect of mode of delivery was seen.

When looking at antibiotic resistance for all the classes with VDnoab as reference (Figure S4), the CSab group showed resistance to several antibiotic classes overall and at week 1 and week 4 including aminoglycoside, beta-lactams and penams, MDR including aminoglycosides, which were drugs administered to the infants during the first week of life. Interestingly, at week 4, CSab showed significantly high resistance to several antibiotic classes with reference to the VDnoab group while the CSnoab group showed none. These differences could be due to the transient nature of the many taxa that colonised during early life in the CSnoab group, which might have reduced in abundance over time. However, in the CSab group, antibiotic exposure in the first 4 days of life resulted in selective pressure that might result in the presence of these ARG-containing bacteria, which were detected at week 4, and then possibly decreased over time (Figure S4).

Additionally, 20 classes of differentially abundant antibiotic resistance classes were detected using MaAsLin2. MaAsLin2 was run using fixed effect as a group variable with each group (VDnoab, CSnoab, CSab) as a reference each time and subject number as a random effect for the overall dataset. Overall, at week 1, and at week 4, the CSab group was strongly (positively) associated with genes conferring resistance to aminoglycoside, beta-lactams, penams, MDR, elfamycin, fluoroquinolone, tetracycline, and macrolide when compared with the other groups. Some of these antibiotic classes such as penams, and aminoglycosides are the same class as those administered to infants in this group during the first 4 days of life (Fig. 4 C). Interestingly, infants in the other two groups, CSnoab and VDnoab also showed positive associations to certain antibiotic classes even at week 1 and week 4 when no antibiotics were administered during the first week of life.

### Strain persistence is increased in vaginal delivery and antibiotic naïve groups

**Fig. 5:**
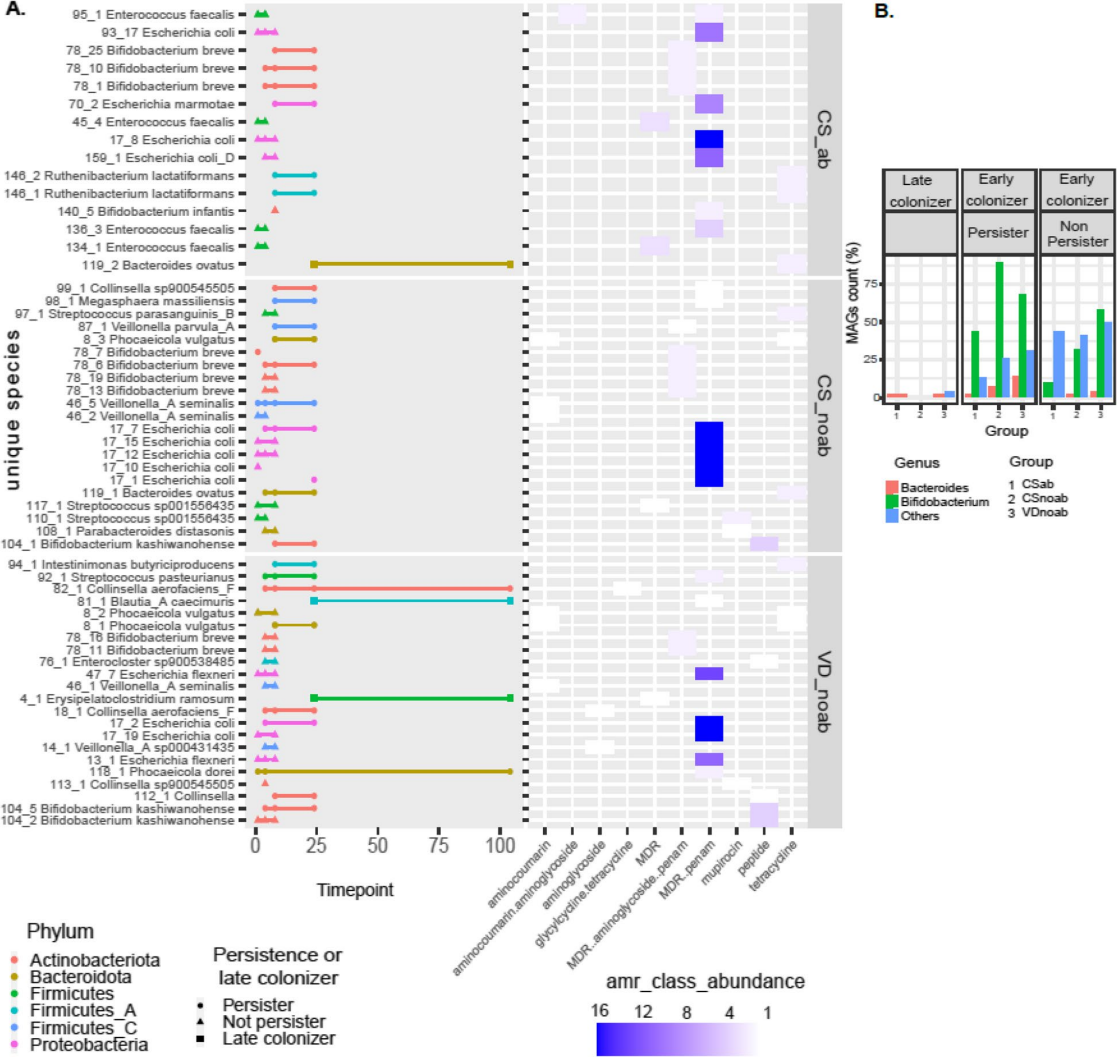
**A** Persistence of strains carrying the top 10 highest abundance of AMR per group and class in infants up to 2 years of age. The right side of the plot depicts abundance as detected by ABRicate in the form of a heatmap. The strains are colour coded based on the phylum to which they belong, as detected by GTDB. **B** Bar plot showing the percent distribution of the genera *Bifidobacterium*, *Bacteroides/Parabacteroides/Phocaeicola*, and others as early colonisers (further divided into persistent colonisers and non-persistent colonisers) and late colonisers for each of the three groups in the study

To understand the prevalence patterns of the resistome in the infants gut, the antibiotic resistance observed due to early life antibiotic exposure was investigated to check if it persists in bacterial strains up to 2 years of age. inStrain was used with MAGs to identify identical strains and examine their persistence in infants in early life. Briefly, bins were dereplicated at 98% whole-genome average nucleotide identity (gANI) to obtain unique strains, which were mapped onto shotgun reads. If the genomic region demonstrated more than 99.99% population-level ANI (popANI) identity in two samples, the strains were considered identical in the two samples. Based on the classification of persistent, non-persistent, and early colonisers (adapted from [43]), a higher number of early colonisers were present in the no-antibiotic groups, with the highest detected in the VDnoab group (Fig. 5B). Further, 98% of the detected strains were early colonisers, of which 45% were non-persistent colonisers. Of the early colonisers, 38% in the CSnoab group, 50% in the VDnoab group, and 48% in the CSab group were non-persistent colonisers (Fig. 5B). The highest number of persistent colonisers that remained until two years of age was in the VDnoab group, which could be due to the higher number of strains inherited maternally by vertical transmission such as those belonging to the Bacteroidota phylum in this group. Overall, due to their abundant appearance in early life in healthy infants and critical role in carbohydrate metabolism in early life, bifidobacteria constituted the genera that contributed to the maximum percentage of persistent colonisers. *Bacteroides* were generally persistent colonisers, with very few non-persistent colonisers, while majorly, Proteobacteria along with some Actinobacteria and Firmicutes were among the top non-persistent colonisers (Figure S5, S6, and S7). Based on the stringent criteria to define strains, we consider that ARG abundance detected within persistent colonisers are the same genes that appeared in early life and persisted until year 2. Investigating the top 10 strains carrying ARGs revealed that all strains in the no-antibiotic groups belonged to the genera *Escherichia*, while those in the CSab group belonged to a mixture of taxa from Gammaproteobacteria, including *Escherichia*, *Klebsiella*, and *Raoultella*; with none persisting up to 2 years of age. A closer look at the top 10 strains per group and antibiotic class showed a wide variety of early colonisers belonging to genera *Escherichia*, *Enterococcus*, *Bifidobacterium*, *Streptococcus*, *Collinsella*, *Phocaeicola*, and *Bacteroides* among others; the majority of these strains were non-persistent colonisers, with most present until week 8 and some until week 24 (Fig. 5A).

### Functional profiles of the microbiota in infants are influenced by antibiotic exposure and age

**Fig. 6:**
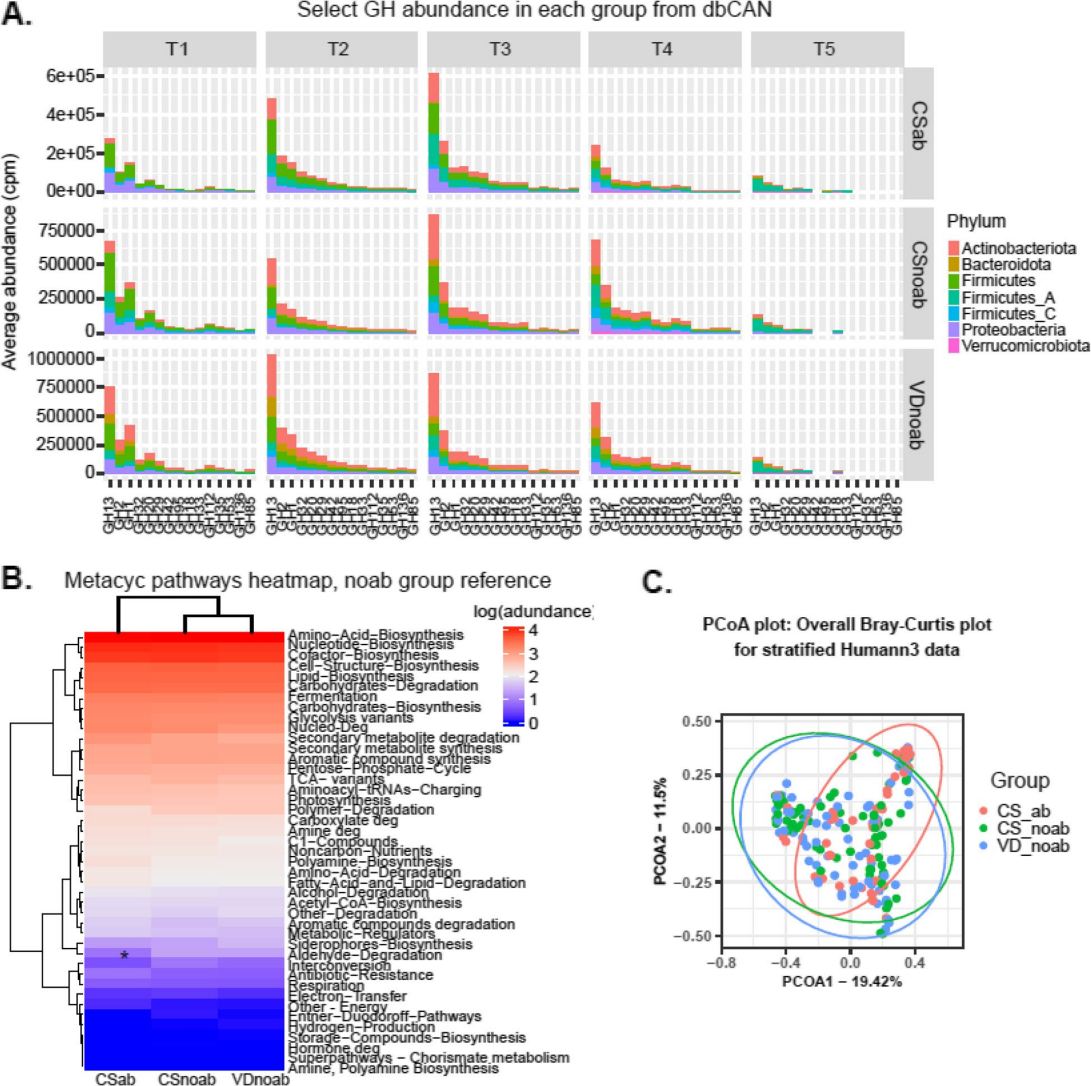
**A** Bar plot depicting percent contribution of each of the detected phyla to cpm normalized abundance of CAZymes important for HMO, GOS, and FOS degradation in infants. The plot presents the percent distribution for each phyla for cpm normalized abundance at each time point for all three groups VDnoab, CSnoab, and CSab. **B** Pathway abundance data for unstratified pathways as detected from HUMAnN3 was categorised into higher MetaCyc pathways. cpm abundance was log-transformed, and a significant difference in abundance between the groups was calculated using the Wilcoxon test with BH using the VDnoab group as a reference. P.adj-values (as denoted by *) < 0.05 are considered significant. **C** PCoA plot using Bray–Curtis distance for stratified pathway abundance data from HUMAnN3, showing distinct clustering of CSab from the other two groups

Differences in microbial composition, relative abundance, and resistome might infer variation in the functional capabilities of the microbiota. Thus, we investigated differences in the functional ability of gut microbiota in the three study groups VDnoab, CSnoab, and CSab from both the MAGs and reads perspectives. Carbohydrate metabolism including HMO and galactooligosaccharides (GOS) degradation are important functions for infant gut microbiota in early life especially when fed breast milk. Overall CAZyme glycoside hydrolase (GH) abundance inferred from the MAGs was significantly higher in the VDnoab group as compared to CSnoab and CSab groups (p.adj =  < 0.0001 and 0.015, respectively; Wilcoxon test using BH method) and CSnoab group had higher GH abundance compared to the CSab group (*p* value = 0.0033) at week 1. At week 4, a significant difference was observed between VDnoab and CSnoab and VDnoab and CSab (*p* value = 3.3e − 06, 9.7e − 06, respectively). CAZyme analysis was also performed for selective GH related to HMO, GOS, and fructooligosaccharides (FOS) degradation enzyme families due to their importance in early life. We observed that the VDnoab group had significantly different overall CAZyme composition (p.adj = 0.012, PERMANOVA) while no other differences were observed otherwise between groups for overall or selective GH composition. Further, CAZyme composition differed significantly based on time with all early time points clustering separately from year 2 for both overall and selective CAZymes (PERMANOVA). Furthermore, delivery mode had a significant impact on the overall and selective GH composition (p.adj = 0.019 and 0.039, respectively, PERMANOVA), while antibiotic use only affected the overall GH composition (p.adj = 0.019). The phyla Actinobacteria and Bacteroidota had the highest percent contribution to GH abundance and it was highest in the VDnoab group. At the same time, Firmicutes and Proteobacteria contributed to a higher percentage in the CS-born infants (Fig. 6A). GH abundance related to HMOs and GOS decreased over time in all three groups, and the VDnoab group had significantly higher abundance than the CSnoab group (*p* value = 0.0035, Wilcoxon test).

Pathway relative abundance from HUMAnN3 was analysed by clustering pathways to higher order MetaCyc pathways. Based on MetaCyc pathways (unstratified) from HUMAnN3 results (Fig. 6B), only aldehyde degradation was observed to be significantly different (lower) in the CSab group compared to the VDnoab group. The antibiotic-treated infants showed a stronger association with pyrimidine nucleotide biosynthesis, ubiquinol (electron carrier biosynthesis), and aromatic compound degradation (toluene, catechol, protocatechuate) pathways, with essential pathways such as glycolysis, phospholipid biosynthesis, nucleic acid processing, carbohydrate degradation, and amino acid biosynthesis increased over time (Table 3). Similarly, NMDS analysis with Bray–Curtis showed a significant difference between week 1 and all later time points, and year 2 and all earlier time points (*p* values < 0.05, Adonis2).

**Table 3:** Table presenting significant *p* values obtained from MaAsLin2 differential abundance analysis for unstratified data from HUMAnN3

Feature	Antibiotic	Coef	*p* value
PWY.7211..superpathway.of.pyrimidine.deoxyribonucleotides.de.novo.biosynthesis	Yes	961.594	0.00019
PPGPPMET.PWY..ppGpp.biosynthesis	Yes	759.515	0.00064
PWY.6545..pyrimidine.deoxyribonucleotides.de.novo.biosynthesis.III	Yes	750.848	0.00117
CATECHOL.ORTHO.CLEAVAGE.PWY..catechol.degradation.to..beta..ketoadipate	Yes	194.333	0.00614
PROTOCATECHUATE.ORTHO.CLEAVAGE.PWY..protocatechuate.degradation.II..ortho.cleavage.pathway	Yes	278.919	0.00382
PWY.5181..toluene.degradation.III..aerobic…via.p.cresol	Yes	218.475	0.00547
PWY.5417..catechol.degradation.III..ortho.cleavage.pathway	Yes	246.549	0.00621
PWY.5431..aromatic.compounds.degradation.via..beta..ketoadipate	Yes	246.549	0.00621
PWY.5855..ubiquinol.7.biosynthesis..prokaryotic	Yes	359.876	0.00680
PWY.5856..ubiquinol.9.biosynthesis..prokaryotic	Yes	359.876	0.00680
PWY.5857..ubiquinol.10.biosynthesis..prokaryotic	Yes	359.876	0.00680
PWY.6318..L.phenylalanine.degradation.IV..mammalian..via.side.chain	Yes	320.314	0.00409
PWY.6562..norspermidine.biosynthesis	Yes	68.1263	0.00334
PWY.6708..ubiquinol.8.biosynthesis..prokaryotic	Yes	359.876	0.00680
PWY.7210..pyrimidine.deoxyribonucleotides.biosynthesis.from.CTP	Yes	638.424	0.00441
PWY0.1297..superpathway.of.purine.deoxyribonucleosides.degradation	Yes	937.509	0.00576
PWY.6182..superpathway.of.salicylate.degradation	Yes	190.901	0.00862
ECASYN.PWY..enterobacterial.common.antigen.biosynthesis	Yes	380.688	0.01017
P185.PWY..formaldehyde.assimilation.III..dihydroxyacetone.cycle	Yes	453.982	0.01145
PWY.7199..pyrimidine.deoxyribonucleosides.salvage	Yes	918.564	0.01226

MaAsLin2 was run using default parameters using min_abundance = 0.00 and min_prevalance = 0.01 with antibiotic as a fixed-effect and sample as random effect

A closer look at the stratified counterpart of pathway abundance data from HUMAnN3 showed study variables including groups, delivery mode, antibiotic exposure in early life, and time-point having an effect on pathway abundance with significant clustering observed between VDnoab and CSab and between CSnoab and CSab groups (Fig. 6C) (pairwise Adonis, PERMANOVA: *p* values = 0.003 and 0.045, respectively, for groups, *p* value = 0.01 by mode of delivery, *p* value = 0.001 by early antibiotic exposure, and *p* value = 0.001 by time point, PERMANOVA). Based on *R*^2^ values from Adonis, time variable and early antibiotic exposure (*R*^2^: 0.15, 0.015, respectively) had the highest influence on separating samples based on pathway abundance; this along with distinct clustering of the CSab group from the no-antibiotic groups point towards the impact of antibiotics in early life on the functionality of the microbiota. Many pathways including those related to sugar metabolism, purine and pyrimidine biosynthesis, and amino acid biosynthesis were associated with the antibiotic-treated group. These differentially abundant pathways were mostly driven by *B. dentium* species, *Ruminococcus*, and *Faecalibacterium* species (Table S3).

## Discussion

We analysed the longitudinal resistome and taxonomic profiles of infants in early-life grouped by early antibiotic use and delivery mode. Results from this study show that the resistome diversity decreases over age from week 1 to year 2, while the microbial diversity increases over time. This is in congruence with previous studies; however, most studies report these profiles only up to year 1 and not many study the resistome profiles as early as week 1. These data also showed the presence of selective antibiotic resistance classes which were administered to the infants in the antibiotic-exposed group at week 1 and week 4. Although the antibiotic in question was only administered in the first 4 days of life, the effect was seen further as age progressed, highlighting the importance of further development of strong stewardship programmes in early life. Additionally, our data also suggest that antibiotic use in early life results in amplification of the resistome profile by selecting MDR bacteria. In this study, antibiotic exposure in early life and mode of delivery were seen to influence microbial diversity and richness between samples longitudinally, with antibiotic exposure having the greatest effect.

A stronger association and higher prevalence of *Bifidobacterium* and *Bacteroides* were detected in vaginally delivered infants, while CS-born infants had higher relative abundance of potential pathogens including *Enterococcus* spp. and species of *Enterobacteriaceae* which was similar to previous reports [7, 19, 21, 22, 54]. CS-born infants are more prone to negative health outcomes such as allergy, asthma, obesity, and diabetes development in later life [55, 56]. Additionally, colonisation patterns of CS infants with bacteria belonging to the category of ESKAPE (*E. faecium*, *S. aureus*, *K. pneumoniae*, *Acinetobacter baumannii*, *Pseudomonas aeruginosa*, and *Enterobacter* species) pathogens are of concern as most bacteria in this group are MDR and are known to act as potential pathogens or are pathobionts [57, 58], thus making any futuristic treatments more difficult to curtail in later life. Many *Bifidobacterium* and Bacteroidota spp. showed lower or delayed colonisation in CS-born infants, with a pronounced effect in the CSab group, which is analogous to previous reports [19, 59, 60], except for *B. dentium* which was seen to have high relative abundance in the CSab group. Recent studies have shown *B. dentium* in high abundance in infants belonging to the intrapartum antibiotic prophylaxis (IAP) group [61, 62] or those fed with donor breast milk [63]. Our result showing high *B. dentium* in the CSab group is in agreement with these reports and further studies are needed to understand the advantageous growth of this *Bifidobacterium* spp. under sub-optimal circumstances. The beneficial and protective roles of *Bifidobacterium* and Bacteroidota spp. in the infant gut, such as fermenting HMOs, maintaining barrier function, enhancing immune responses, colonisation resistance, and competing for nutrients against pathogens are well established [60–62, 64–67]. Decreased colonisation of these beneficial taxa is associated with adverse outcomes such as necrotising enterocolitis and late-onset sepsis in the preterm population [68, 69], thus demanding further attention for studies in infants born by CS and exposed to antibiotics.

A diverse range of ARGs and MDR bacteria was detected in all three groups, with decreasing ARG diversity seen over time. The most common antibiotic classes observed were MDR, which includes resistance to penams, fluoroquinolones, peptides, aminocoumarin, aminoglycosides, and tetracyclines. This is in line with many reports suggesting the infant gut microbiota is a reservoir for ARGs [11, 12, 70]. The top abundance of ARGs was detected in *Escherichia*, *Klebsiella*, *Enterobacter*, *Bifidobacterium*, *Raoultella*, and other Proteobacteria and Firmicutes species, with the highest association of antibiotic classes to the CSab group. Our findings are consistent with previous studies that report a high abundance of ARGs in several taxa belonging to Gammaproteobacteria [12, 17, 71–73]. The CSab group also showed the presence of ARGs and stronger associations with classes of antibiotics administered to the infants in this group, clustering separately from the no-antibiotic groups, further pointing towards the fact that antibiotic exposure contributed to ARG abundance more than the delivery mode.

This study found a higher frequency of ARGs in the CSab group, followed by the CSnoab and VDnoab groups, primarily due to this group’s antibiotic exposure in early life. This can be attributed to the fact that several commensals and pathobionts harbour a set of ARGs, which could be inherited or transferred by horizontal gene transfer in the highly populated environment in the gut. Additionally, the use of antibiotics in early life alters the microbial composition and results in the selection of bacteria whose ARG reservoir includes genes capable of conferring resistance to the class of antibiotic in question. The selection of such bacteria resistant to the antibiotic in question may possess several other ARGs leading to the presence and detection of MDR bacteria. These pathobionts can switch to their opportunistic pathogenic side and can lead to infections that are difficult to treat due to the plethora of ARGs they contain. Disturbed microbial colonisation due to delivery mode and early antibiotic exposure can lead to colonisation by more hospital-acquired strains. These nosocomial strains are enriched with a diverse resistome profile [74–77]. Furthermore, the high frequency of ARGs found in this study was associated with their high abundance in Proteobacteria. Increased abundance of specific taxa in this group (such as some *Klebsiella*, *Enterobacter*, *Raoultella* species) is reported to be associated with several functional variations and difficult-to-treat infections [78–81]. The establishment of such strains from infancy in the gut can lead to complications in treatment in later life. Highly regulated antibiotic stewardship programmes are thus necessary along with probable therapeutic administration of probiotics where possible. Due to the stringent parameters used to define a strain, we were able to examine the persistence of resistance in the strains and observed that the top 10 strains per class and group showed very little to no persistence up to two years of age. Strains that carried the highest ARG abundance mostly belonged to the genera *Enterococcus*, *Escherichia*, *Veillonella*, *Streptococcus*, *Bifidobacterium*, and *Bacteroidetes*. Moreover, *Bifidobacterium* strains and Bacteroidota strains were observed to be major persistent colonisers, which sustain up to two years of age with more persistence observed in the non-antibiotic groups with the highest in the vaginally delivered infants.

Though overall GH abundance was significantly different and higher in the VDnoab group and lowest in the CSab group at week 1 and week 4, this difference was not prominent when selective GH related to HMO, FOS, and GOS enzyme families were considered. Further, the percent contribution of various phyla to select GH functions was observed to vary between groups, prominently seen in early time points. This could be because functions like HMO degradation are essential and redundant in early life and colonisation is not solely dependent on feeding habits, mode of delivery, or antibiotic exposure. While *Bifidobacterium* and *Bacteroides* are recognised as proficient HMO degraders [82, 83], their scarcity or non-existence in infants does not negate the critical role of HMO degradation, which can still be performed by other bacterial species present in these infants. Similarly, unstratified metaCyC pathways did not show significant differences when the CS-born infant groups were compared to the VDnoab group while based on stratified pathway abundance data CSab was significantly different from the other two groups. This could be because the specific microbial composition of each group contributes to similar functional attributes. Furthermore, strong associations of perinatal factors such as antibiotic treatment with aromatic compound degradation pathways, amino acid biosynthesis, cofactor and coenzyme biosynthesis, nucleotide biosynthesis (both purine and pyrimidine), and carbohydrate degradation pathways were observed. Similar associations such as increased nucleotide biosynthesis pathways and carbohydrate degradation were reported earlier in antibiotic-treated groups [84, 85]. Such changes in metabolic potential can affect children’s health in later life, and detailed studies will help understand these changes and their influence.

Some strengths of this study include examining several factors such as delivery mode, antibiotic use in early life, and age on the dynamic nature of microbial colonisation pattern with read- and assembly-based approaches using shotgun sequencing. We also inspected and observed the taxa in infants that act as persistent colonisers and colonise the gut up to 2 years of age. This study was limited due to a small cohort of 45 infants and limited sample numbers between time points. Further, clinical metadata of antibiotic exposure relating to all time points of sample collection or similar data from the mothers were missing, which narrows down the hypotheses that could be validated in the study. Thus, future studies with detailed clinical metadata and more time points with smaller intervals will help unfold the complex relationship between study variables, microbial and resistome profile and functionality of the microbiota.

## Conclusion

This study highlights the effects of antibiotic exposure in early life and mode of delivery on infant gut microbiota in a longitudinal setting. Treatment with a combination of benzylpenicillin and gentamicin in early life has a stronger influence over the type and order of microbial colonisation in infants in early life than delivery mode. Such disturbed colonisation, along with a diverse resistance reservoir impact the functional capability of the infant gut microbiota, including but not limited to the impairment that can be caused to other crucial functional capacities such as colonisation resistance, metabolism, maintaining gut permeability, and a healthy immune response. Thus, more studies with larger cohorts are needed to better understand ARGs’ persistence and impact on microbial functional capability with more certainty. Moreover, new alternatives/strategies need to be developed and used where needed to restore the microbial ecosystem, maintain a healthy microenvironment, restore their functional powers, and reduce the use of prophylactic antibiotics during the crucial infancy stage.

### Supplementary Information

**Additional file 1: Figure S1.** A. Microbial diversity of the infant gut as measured using Shannon diversity index between all three groups at each time-point in this study. Significant difference in diversity was observed between CSab and Csnoab at week 1 and 24. B. Beta-diversity using unweighted Unifrac distance as depicted using PCoA at each time point. **Figure S2.** A. Relative abundance plot showing phylum level distribution in all the study groups at each time-point. B. Percent variance for each time point plotted using R2 values as generated from PERMANOVA for Antibiotic and Mode of delivery variables in the study using unweighted Unifrac distance. C. Plots shows differential abundance analysis using Songbird with CSnoab group as reference run using the formula C(Group, Treatment('CSnoab')). Plot on the left side corresponds to VDnoab group as treatment while that on right depicts CSab group as treatment against the reference group. In both cases negative value (Blue bars) represent association to the reference group (here CSnoab) while positive values (red bars) represent association to the treatment group; here VDnoab (on left plot) and CSab (right side plot). **Figure S3.** A. Plot showing top 10 species possessing highest abundance (cpm normalised) of ARGs as detected using ABRicate. B. Beta-diversity computed for ARG distribution from RGI using Bray–Curtis distance metrics and plotted using PCoA shows distinct clustering between 1. Groups, 2. Samples based on antibiotic exposure in first four days of life and 3. study time-points. **Figure S4.** All antibiotic classes as detected using RGI, depicting correlation of each group to AMR class abundance at A. Overall, B. week 1 and C. at week 4. Fluoroquinolone is abbreviated to FQ in the above plot. *P*-values < 0.05 were considered significant and were generated using Wilcoxon test with VDnoab group as reference. **Figure S5.** Persistence of all unique strains as observed from inStrains for VDnoab group. Right side of the plot depicts abundance of ARGs as detected by ABRicate in the form of heatmap. Left side of the plot depicts persistence of strains up to two years of age. The strains are colour coded based on the phylum which they belong to, as detected by GTDB. **Figure S6.** Persistence of all unique strains as observed from inStrains for CSnoab group. Right side of the plot depicts abundance of ARGs as detected by ABRicate in the form of heatmap. Left side of the plot depicts persistence of strains up to two years of age. The strains are colour coded based on the phylum which they belong to, as detected by GTDB. **Figure S7.** Persistence of all unique strains as observed from inStrains for CSab group. Right side of the plot depicts abundance of ARGs as detected by ABRicate in the form of heatmap. Left side of the plot depicts persistence of strains up to two years of age. The strains are colour coded based on the phylum which they belong to, as detected by GTDB. **Table S1.** Table shows possible contaminants as flagged from inStrains results. **Table S2. **Table shows coefficients obtained by running differential abundance analysis using Songbird with CSnoab group as reference run using the formula C(Group, Treatment('CSnoab')). **Table S3.** Table presenting significant p-values obtained from MaAsLin2 differential abundance analysis for stratified data from HUMAnN3. MaAsLin2 was run using Antibiotic as Fixed effect and Sample as random effect with default parameters of min_abundance = 0.00 and min_prevalance = 0.01.

## Data Availability

The metagenomics data generated as a part of this study is available on NCBI with the SRA accession number PRJNA971895, available here: https://www.ncbi.nlm.nih.gov/sra/PRJNA971895.
