# Supplementary material for: Early life exposure of infants to benzylpenicillin and gentamicin is associated with a persistent amplification of the gut resistome
Source: Microbiome. 2024 Feb 3;12:19. doi: 10.1186/s40168-023-01732-6 (PMC10837951; doi:10.1186/s40168-023-01732-6)
Supplement: Supplementary file 2 — Additional file 1: Figure S1. A. Microbial diversity of the infant gut as measured using Shannon diversity index between all three groups at each time-point in this study. Significant difference in diversity was observed between CSab and Csnoab at week 1 and 24. B. Beta-diversity using unweighted Unifrac distance as depicted using PCoA at each time point. Figure S2. A. Relative abundance plot showing phylum level distribution in all the study groups at each time-point. B. Percent variance for each time point plotted using R2 values as generated from PERMANOVA for Antibiotic and Mode of delivery variables in the study using unweighted Unifrac distance. C. Plots shows differential abundance analysis using Songbird with CSnoab group as reference run using the formula C(Group, Treatment('CSnoab')). Plot on the left side corresponds to VDnoab group as treatment while that on right depicts CSab group as treatment against the reference group. In both cases negative value (Blue bars) represent association to the reference group (here CSnoab) while positive values (red bars) represent association to the treatment group; here VDnoab (on left plot) and CSab (right side plot). Figure S3. A. Plot showing top 10 species possessing highest abundance (cpm normalised) of ARGs as detected using ABRicate. B. Beta-diversity computed for ARG distribution from RGI using Bray–Curtis distance metrics and plotted using PCoA shows distinct clustering between 1. Groups, 2. Samples based on antibiotic exposure in first four days of life and 3. study time-points. Figure S4. All antibiotic classes as detected using RGI, depicting correlation of each group to AMR class abundance at A. Overall, B. week 1 and C. at week 4. Fluoroquinolone is abbreviated to FQ in the above plot. P-values < 0.05 were considered significant and were generated using Wilcoxon test with VDnoab group as reference. Figure S5. Persistence of all unique strains as observed from inStrains for VDnoab group. Right side of the p [file 40168_2023_1732_MOESM1_ESM.docx]

Supplementary figures:

Figure S1:


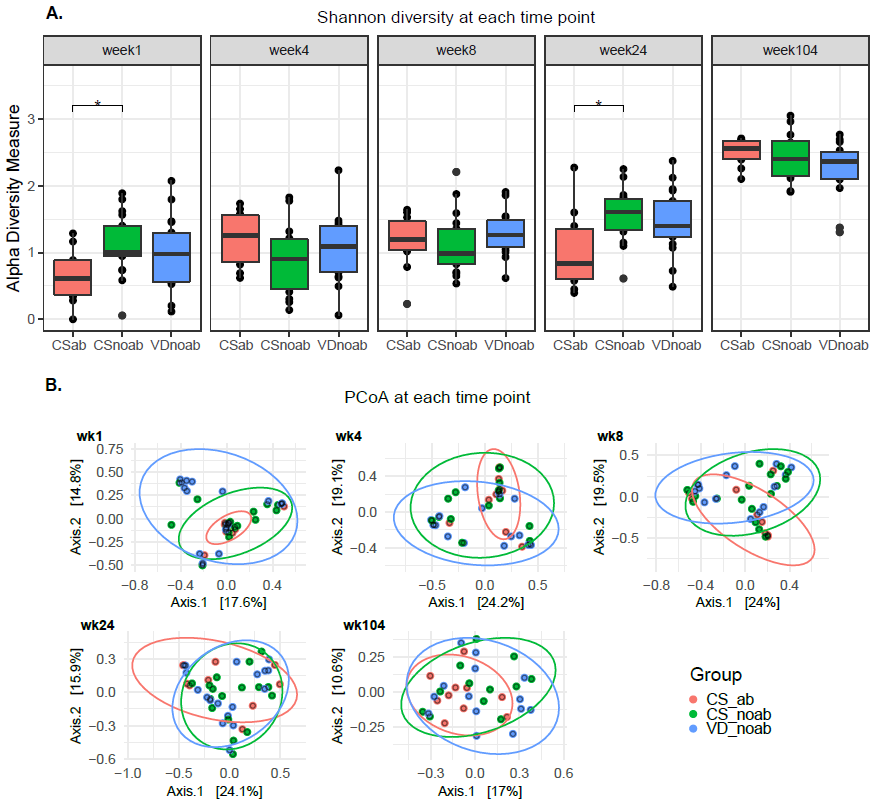


Figure S1: A. Microbial diversity of the infant gut as measured using Shannon diversity index between all three groups at each time-point in this study. Significant difference in diversity was observed between CSab and Csnoab at week 1 and 24. B. Beta diversity using unweighted Unifrac distance as depicted using PCoA at each time point.

Figure S2:


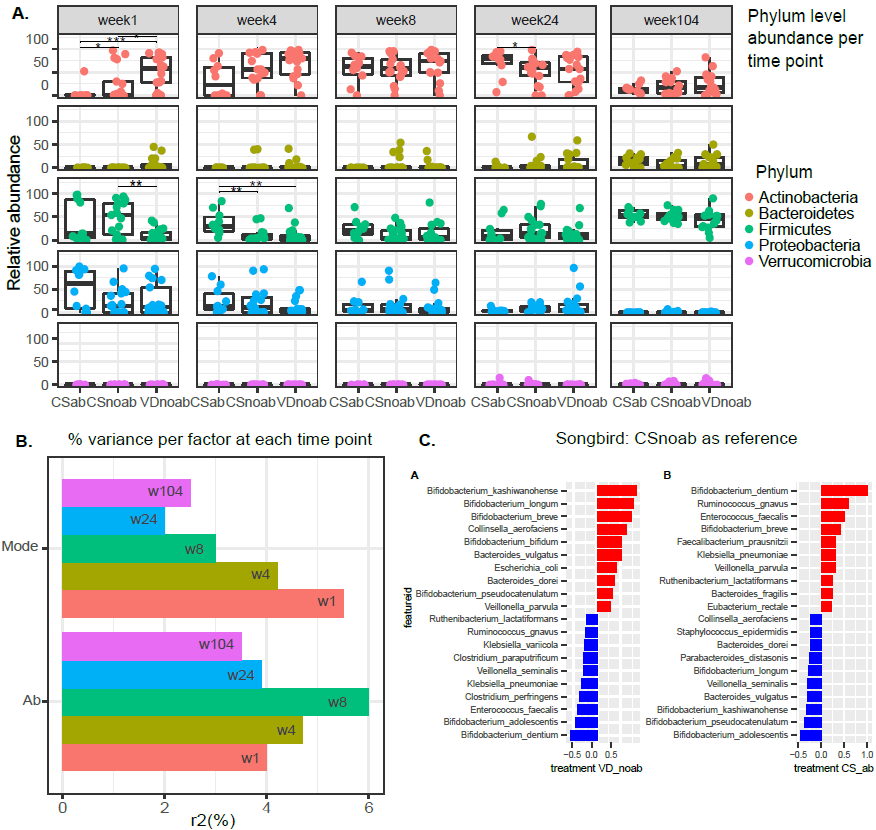


Figure S2: A. Relative abundance plot showing phylum level distribution in all the study groups at each time-point. B. Percent variance for each time point plotted using R2 values as generated from PERMANOVA for Antibiotic and Mode of delivery variables in the study using unweighted Unifrac distance. C. Plots shows differential abundance analysis using Songbird with CSnoab group as reference run using the formula C(Group, Treatment('CSnoab')). Plot on the left side corresponds to VDnoab group as treatment while that on right depicts CSab group as treatment against the reference group. In both cases negative value (Blue bars) represent association to the reference group (here CSnoab) while positive values (red bars) represent association to the treatment group; here VDnoab (on left plot) and CSab (right side plot).

Figure S3:


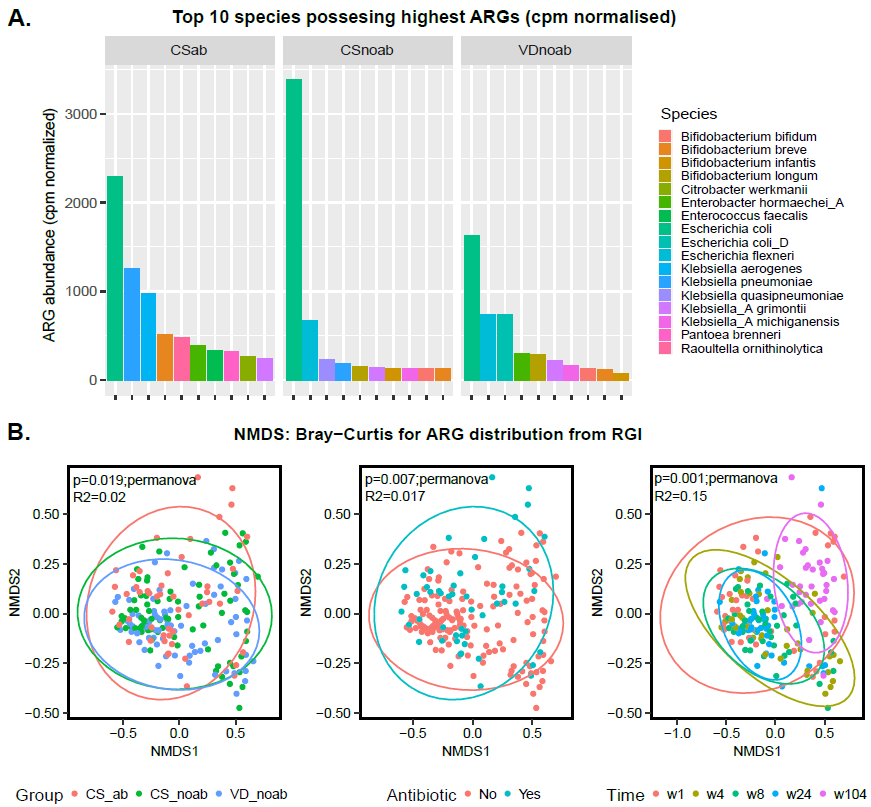


Figure S3: A. Plot showing top 10 species possessing highest abundance (cpm normalised) of ARGs as detected using ABRicate. B. Beta diversity computed for ARG distribution from RGI using Bray-Curtis distance metrics and plotted using PCoA shows distinct clustering between 1. Groups, 2. Samples based on antibiotic exposure in first four days of life and 3. study time-points.

Figure S4:


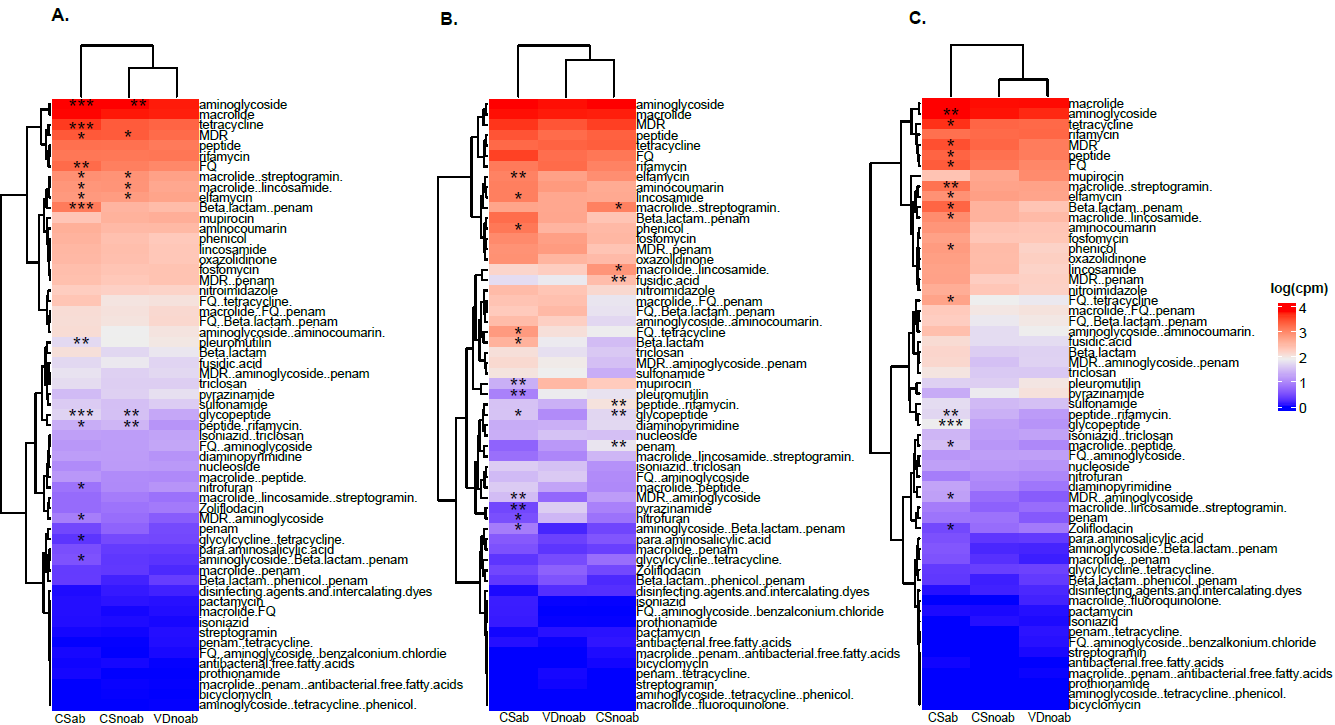


Figure S4: All antibiotic classes as detected using RGI, depicting correlation of each group to AMR class abundance at A. Overall, B. week 1 and C. at week 4. Fluoroquinolone is abbreviated to FQ in the above plot. P-values < 0.05 were considered significant and were generated using Wilcoxon test with VDnoab group as reference.

Figure S5:


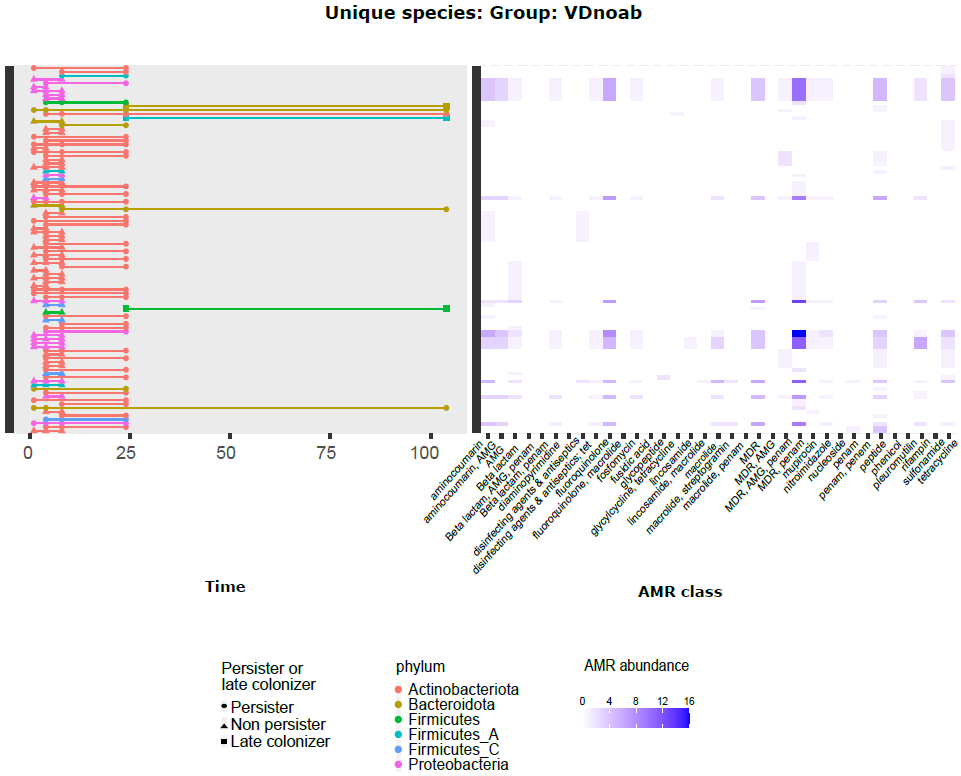


Figure S5: Persistence of all unique strains as observed from inStrains for VDnoab group. Right side of the plot depicts abundance of ARGs as detected by ABRicate in the form of heatmap. Left side of the plot depicts persistence of strains up to two years of age. The strains are colour coded based on the phylum which they belong to, as detected by GTDB.

Figure S6


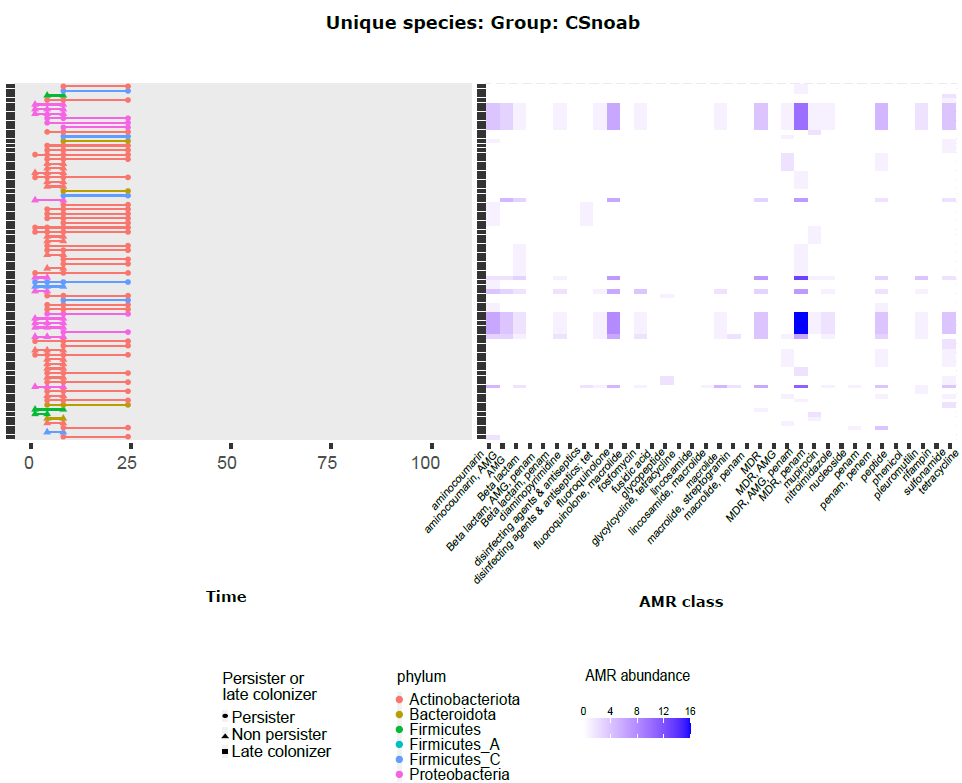


Figure S6: Persistence of all unique strains as observed from inStrains for CSnoab group. Right side of the plot depicts abundance of ARGs as detected by ABRicate in the form of heatmap. Left side of the plot depicts persistence of strains up to two years of age. The strains are colour coded based on the phylum which they belong to, as detected by GTDB.

Figure S7:


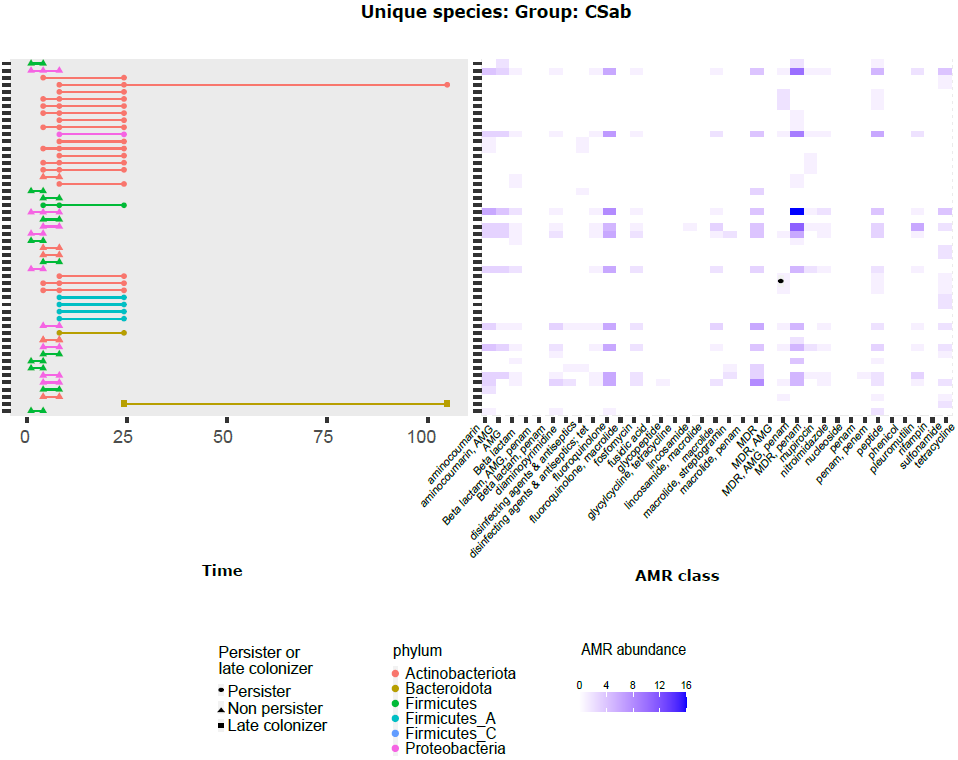


Figure S7: Persistence of all unique strains as observed from inStrains for CSab group. Right side of the plot depicts abundance of ARGs as detected by ABRicate in the form of heatmap. Left side of the plot depicts persistence of strains up to two years of age. The strains are colour coded based on the phylum which they belong to, as detected by GTDB.

Supplementary tables:

Table S1: Table shows possible contaminants as flagged from inStrains results.

| **cluster** | **sample** | **genome** | **Individual** | **Remark** | **Contamination** | **Plate #** |
| --- | --- | --- | --- | --- | --- | --- |
| 140_5 | M15w4.sorted.bam | M20w24_bin.1.fna | S15 | Multiple S15 and S20 clusters. Siblings? |  | plate 2 |
| 140_5 | M15w8.sorted.bam | M20w24_bin.1.fna | S15 |  |  | plate 2 |
| 140_5 | M20w24.sorted.bam | M20w24_bin.1.fna | S20 |  |  | plate 2 |
| 140_5 | M25w4.sorted.bam | M20w24_bin.1.fna | S25 |  |  | plate 2 |
| 140_5 | M25w8.sorted.bam | M20w24_bin.1.fna | S25 |  |  | plate 2 |
| 152_1 | M15w8.sorted.bam | M31w8_bin.2.fna | S15 |  |  | plate 2 |
| 152_1 | M20w8.sorted.bam | M31w8_bin.2.fna | S20 |  |  | plate 2 |
| 122_4 | I08w24.sorted.bam | M08y2_bin.4.fna | S08 | Are S08 and S18 siblings? There's a lot of clusters shared by the two |  | plate 3 |
| 122_4 | I08w4.sorted.bam | M08y2_bin.4.fna | S08 |  |  | plate 2 |
| 122_4 | I08w8.sorted.bam | M08y2_bin.4.fna | S08 |  |  | plate 2 |
| 122_4 | I18w4.sorted.bam | M08y2_bin.4.fna | S18 |  |  | plate 2 |
| 131_1 | I08w24.sorted.bam | M13w24_bin.5.fna | S08 |  |  | plate 3 |
| 131_1 | I08w4.sorted.bam | M13w24_bin.5.fna | S08 |  |  | plate 2 |
| 131_1 | I08w8.sorted.bam | M13w24_bin.5.fna | S08 |  |  | plate 2 |
| 131_1 | I18w4.sorted.bam | M13w24_bin.5.fna | S18 |  |  | plate 2 |
| 140_1 | I08w24.sorted.bam | M20w24_bin.1.fna | S08 |  |  | plate 3 |
| 140_1 | I08w4.sorted.bam | M20w24_bin.1.fna | S08 |  |  | plate 2 |
| 140_1 | I08w8.sorted.bam | M20w24_bin.1.fna | S08 |  |  | plate 2 |
| 140_1 | I18w4.sorted.bam | M20w24_bin.1.fna | S18 |  |  | plate 2 |
| 26_1 | I08w24.sorted.bam | I18w24_bin.2.fna | S08 |  |  | plate 3 |
| 26_1 | I08w4.sorted.bam | I18w24_bin.2.fna | S08 |  |  | plate 2 |
| 26_1 | I08w8.sorted.bam | I18w24_bin.2.fna | S08 |  |  | plate 2 |
| 26_1 | I18w4.sorted.bam | I18w24_bin.2.fna | S18 |  |  | plate 2 |
| 55_22 | I08w24.sorted.bam | I90w1_bin.7.fna | S08 |  |  | plate 2 |
| 55_22 | I08w4.sorted.bam | I90w1_bin.7.fna | S08 |  |  | plate 3 |
| 55_22 | I08w8.sorted.bam | I90w1_bin.7.fna | S08 |  |  | plate 2 |
| 55_22 | I18w4.sorted.bam | I90w1_bin.7.fna | S18 |  |  | plate 2 |
| 147_17 | I08w4.sorted.bam | M29w8_bin.2.fna | S08 |  |  | plate 2 |
| 147_17 | I08w8.sorted.bam | M29w8_bin.2.fna | S08 |  |  | plate 2 |
| 147_17 | I18w4.sorted.bam | M29w8_bin.2.fna | S18 |  |  | plate 2 |
| 17_1 | I64w8.sorted.bam | I159W4_bin.1.fna | S64 | Multiple S92 and S64 clusters (2). Siblings, contamination? | Different plates | plate 2 |
| 17_1 | I92w24.sorted.bam | I159W4_bin.1.fna | S92 |  | Different plates | plate 3 |
| 17_1 | IM92w8.sorted.bam | I159W4_bin.1.fna | S92 |  | Different plates | plate 1 |
| 93_1 | I64w8.sorted.bam | IM161w4_bin.5.fna | S64 |  | Different plates | plate 2 |
| 93_1 | I92w24.sorted.bam | IM161w4_bin.5.fna | S92 |  | Different plates | plate 3 |
| 93_1 | IM92w8.sorted.bam | IM161w4_bin.5.fna | S92 |  | Different plates | plate 1 |
| 37_1 | I57w1.sorted.bam | I57w1_bin.4.fna | S57 | I157w1 and M15w4 are not on the same plate. Contamination before plating possible? Siblings? | Different plates | plate 3 |
| 37_1 | M15w4.sorted.bam | I57w1_bin.4.fna | S15 |  | Different plates | plate 2 |
| 79_26 | I159W24.sorted.bam | IM143w8_bin.4.fna | S159 | Contamination? | Different plates | plate 4 |
| 79_26 | M43y2.sorted.bam | IM143w8_bin.4.fna | S43 |  | Different plates | plate 2 |
| 79_27 | I10w24.sorted.bam | IM143w8_bin.4.fna | S10 | Contamination of S10? | Different plates | plate 3 |
| 79_27 | M15w24.sorted.bam | IM143w8_bin.4.fna | S15 |  | Different plates | plate 2 |
| 79_27 | M15w8.sorted.bam | IM143w8_bin.4.fna | S15 |  | Different plates | plate 2 |
| 79_27 | M15y2.sorted.bam | IM143w8_bin.4.fna | S15 |  | Different plates | plate 2 |
| 2_1 | I144w24.sorted.bam | I01w24_bin.10.fna | S144 | Contamination of S144? | Different plates | plate 3 |
| 2_1 | IM01w4.sorted.bam | I01w24_bin.10.fna | S01 |  | Different plates | plate 1 |
| 2_1 | IM01w8.sorted.bam | I01w24_bin.10.fna | S01 |  | Different plates | plate 1 |

Table S2: Table shows coefficients obtained by running differential abundance analysis using Songbird with CSnoab group as reference run using the formula C(Group, Treatment('CSnoab')).

| featureid | Treatment: CSab | Treatment: VDnoab |
| --- | --- | --- |
| s__*Bifidobacterium_adolescentis* | -0.461624786 | -0.44888641 |
| s__*Bifidobacterium_pseudocatenulatum* | -0.376452371 | 0.334038301 |
| s__*Bifidobacterium_kashiwanohense* | -0.319553137 | 0.823212488 |
| s__*Bacteroides_vulgatus* | -0.304268077 | 0.513280137 |
| s__*Veillonella_seminalis* | -0.29253386 | -0.280086697 |
| s__*Bifidobacterium_longum* | -0.285144448 | 0.768663629 |
| s__*Parabacteroides_distasonis* | -0.264707908 | 0.119381948 |
| s__*Bacteroides_dorei* | -0.243102014 | 0.363738581 |
| s__*Staphylococcus_epidermidis* | -0.239515215 | -0.04300128 |
| s__*Collinsella_aerofaciens* | -0.227144569 | 0.618961438 |
| s__*Escherichia_coli* | -0.218357757 | 0.41331832 |
| s__*Staphylococcus_hominis* | -0.188115552 | -0.132084594 |
| s__*Veillonella_atypica* | -0.185183957 | 0.0767114 |
| s__*Streptococcus_parasanguinis* | -0.185049698 | -0.192258553 |
| s__*Clostridium_perfringens* | -0.173089847 | -0.374569745 |
| s__*Ruminococcus_lactaris* | -0.146253064 | -0.169122056 |
| s__*Clostridium_paraputrificum* | -0.141725525 | -0.278720648 |
| s__*Veillonella_dispar* | -0.140300497 | -0.192313359 |
| s__*Roseburia_faecis* | -0.139978066 | -0.110566572 |
| s__*Eubacterium_hallii* | -0.139586702 | -0.012941704 |
| s__*Dorea_longicatena* | -0.118079945 | -0.164423929 |
| s__*Roseburia_inulinivorans* | -0.116796508 | -0.209173308 |
| s__*Fusicatenibacter_saccharivorans* | -0.091681316 | -0.032283531 |
| s__*Ruminococcus_bromii* | -0.073511198 | -0.13761212 |
| s__*Bacteroides_ovatus* | -0.060816064 | -0.101639614 |
| s__*Flavonifractor_plautii* | -0.056162164 | -0.210859493 |
| s__*Ruminococcus_torques* | -0.046746746 | -0.085351364 |
| s__*Bacteroides_uniformis* | -0.03068395 | 0.068745299 |
| s__*Akkermansia_muciniphila* | -0.027762488 | -0.154325889 |
| s__*Eubacterium_eligens* | 0.046054617 | -0.109816806 |
| s__*Erysipelatoclostridium_ramosum* | 0.06502445 | 0.209913655 |
| s__*Klebsiella_variicola* | 0.073789433 | -0.26101406 |
| s__*Lactobacillus_paragasseri* | 0.111511782 | -0.180475161 |
| s__*Streptococcus_salivarius* | 0.125989512 | -0.155585305 |
| s__*Clostridium_innocuum* | 0.126462594 | -0.117592559 |
| s__*Blautia_wexlerae* | 0.12969543 | 0.108885272 |
| s__*Streptococcus_vestibularis* | 0.13077648 | -0.109347896 |
| s__*Bifidobacterium_bifidum* | 0.156286195 | 0.518355413 |
| s__*Anaerostipes_hadrus* | 0.175960198 | 0.089932545 |
| s__*Eubacterium_rectale* | 0.212511137 | -0.119487659 |
| s__*Bacteroides_fragilis* | 0.24164857 | 0.090509995 |
| s__*Ruthenibacterium_lactatiformans* | 0.251872912 | -0.220478044 |
| s__*Veillonella_parvula* | 0.300057068 | 0.290145083 |
| s__*Klebsiella_pneumoniae* | 0.315716937 | -0.316212238 |
| s__*Faecalibacterium_prausnitzii* | 0.315744594 | -0.010124699 |
| s__*Bifidobacterium_breve* | 0.408126071 | 0.731155856 |
| s__*Enterococcus_faecalis* | 0.50914903 | -0.409636241 |
| s__*Ruminococcus_gnavus* | 0.588117793 | -0.232703791 |
| s__*Bifidobacterium_dentium* | 1.009432629 | -0.566254036 |

Table S3: Table presenting significant p-values obtained from MaAsLin2 differential abundance analysis for stratified data from HUMAnN3. MaAsLin2 was run using Antibiotic as Fixed effect and Sample as random effect with default parameters of min_abundance = 0.00 and min_prevalance=0.01.

| feature | Antibiotic | coef | pval |
| --- | --- | --- | --- |
| ARGSYNBSUB.PWY..L.arginine.biosynthesis.II..acetyl.cycle..g__Bifidobacterium.s__*Bifidobacterium_dentium* | yes | 398.3997 | 0.0006 |
| BRANCHED.CHAIN.AA.SYN.PWY..superpathway.of.branched.amino.acid.biosynthesis.g__Bifidobacterium.s__*Bifidobacterium_dentium* | yes | 455.515 | 0.0010 |
| COA.PWY.1..coenzyme.A.biosynthesis.II..mammalian..g__Bifidobacterium.s__*Bifidobacterium_dentium* | yes | 427.7017 | 0.0007 |
| COA.PWY..coenzyme.A.biosynthesis.I.g__Bifidobacterium.s__*Bifidobacterium_dentium* | yes | 386.625 | 0.0007 |
| DTDPRHAMSYN.PWY..dTDP.L.rhamnose.biosynthesis.I.g__Bifidobacterium.s__*Bifidobacterium_dentium* | yes | 488.6735 | 0.0009 |
| HISTSYN.PWY..L.histidine.biosynthesis.g__Bifidobacterium.s__*Bifidobacterium_dentium* | yes | 380.7538 | 0.0009 |
| ILEUSYN.PWY..L.isoleucine.biosynthesis.I..from.threonine..g__Bifidobacterium.s*__Bifidobacterium_dentium* | yes | 473.6178 | 0.0011 |
| NONMEVIPP.PWY..methylerythritol.phosphate.pathway.I.g__Bifidobacterium.s__*Bifidobacterium_dentium* | yes | 397.1707 | 0.0008 |
| OANTIGEN.PWY..O.antigen.building.blocks.biosynthesis..E..coli..g__Bifidobacterium.s__*Bifidobacterium_dentium* | yes | 396.0303 | 0.0010 |
| P124.PWY..Bifidobacterium.shunt.g__Bifidobacterium.s__*Bifidobacterium_dentium* | yes | 401.8314 | 0.0009 |
| PWY.2942..L.lysine.biosynthesis.III.g__Bifidobacterium.s__*Bifidobacterium_dentium* | yes | 488.1304 | 0.0007 |
| PWY.3001..superpathway.of.L.isoleucine.biosynthesis.I.g__Bifidobacterium.s__*Bifidobacterium_dentium* | yes | 440.3852 | 0.0008 |
| PWY.3841..folate.transformations.II.g__Bifidobacterium.s__*Bifidobacterium_dentium* | yes | 375.2278 | 0.0006 |
| PWY.4242..pantothenate.and.coenzyme.A.biosynthesis.III.g__Bifidobacterium.s__*Bifidobacterium_dentium* | yes | 389.7976 | 0.0007 |
| PWY.5097..L.lysine.biosynthesis.VI.g__Bifidobacterium.s__*Bifidobacterium_dentium* | yes | 429.1196 | 0.0008 |
| PWY.5103..L.isoleucine.biosynthesis.III.g__Bifidobacterium.s__*Bifidobacterium_dentium* | yes | 442.9995 | 0.0009 |
| PWY.5384..sucrose.degradation.IV..sucrose.phosphorylase..g__Bifidobacterium.s__*Bifidobacterium_dentium* | yes | 517.8944 | 0.0007 |
| PWY.5659..GDP.mannose.biosynthesis.g__Bifidobacterium.s__*Bifidobacterium_dentium* | yes | 390.9609 | 0.0007 |
| PWY.5686..UMP.biosynthesis.g__Bifidobacterium.s__*Bifidobacterium_dentium* | yes | 536.4589 | 0.0007 |
| PWY.6121..5.aminoimidazole.ribonucleotide.biosynthesis.I.g__Bifidobacterium.s__*Bifidobacterium_dentium* | yes | 340.0982 | 0.0008 |
| PWY.6122..5.aminoimidazole.ribonucleotide.biosynthesis.II.g__Bifidobacterium.s__*Bifidobacterium_dentium* | yes | 369.1303 | 0.0008 |
| PWY.6123..inosine.5..phosphate.biosynthesis.I.g__Bifidobacterium.s__*Bifidobacterium_dentium* | yes | 365.3022 | 0.0007 |
| PWY.6124..inosine.5..phosphate.biosynthesis.II.g__Bifidobacterium.s__*Bifidobacterium_dentium* | yes | 344.4034 | 0.0007 |
| PWY.6151..S.adenosyl.L.methionine.cycle.I.g__Bifidobacterium.s__*Bifidobacterium_dentium* | yes | 430.8586 | 0.0008 |
| PWY.6277..superpathway.of.5.aminoimidazole.ribonucleotide.biosynthesis.g__Bifidobacterium.s__*Bifidobacterium_dentium* | yes | 369.1303 | 0.0008 |
| PWY.6385..peptidoglycan.biosynthesis.III..mycobacteria..g__Bifidobacterium.s__*Bifidobacterium_dentium* | yes | 363.3126 | 0.0007 |
| PWY.6387..UDP.N.acetylmuramoyl.pentapeptide.biosynthesis.I..meso.diaminopimelate.containing..g__Bifidobacterium.s__*Bifidobacterium_dentium* | yes | 342.8993 | 0.0007 |
| PWY.6737..starch.degradation.V.g__Bifidobacterium.s__*Bifidobacterium_dentium* | yes | 538.29 | 0.0008 |
| PWY.7111..pyruvate.fermentation.to.isobutanol..engineered..g__Bifidobacterium.s__*Bifidobacterium_dentium* | yes | 474.6266 | 0.0010 |
| PWY.7219..adenosine.ribonucleotides.de.novo.biosynthesis.g__Bifidobacterium.s__*Bifidobacterium_dentium* | yes | 438.3994 | 0.0007 |
| PWY.7221..guanosine.ribonucleotides.de.novo.biosynthesis.g__Bifidobacterium.s__*Bifidobacterium_dentium* | yes | 611.7984 | 0.0007 |
| PWY.7234..inosine.5..phosphate.biosynthesis.III.g__Bifidobacterium.s__*Bifidobacterium_dentium* | yes | 365.5587 | 0.0007 |
| PWY.724..superpathway.of.L.lysine..L.threonine.and.L.methionine.biosynthesis.II.g__Bifidobacterium.s__*Bifidobacterium_dentium* | yes | 413.5308 | 0.0007 |
| THRESYN.PWY..superpathway.of.L.threonine.biosynthesis.g__Bifidobacterium.s__*Bifidobacterium_dentium* | yes | 424.5996 | 0.0007 |
| UDPNAGSYN.PWY..UDP.N.acetyl.D.glucosamine.biosynthesis.I.g__Bifidobacterium.s__*Bifidobacterium_dentium* | yes | 369.8979 | 0.0010 |
| VALSYN.PWY..L.valine.biosynthesis.g__Bifidobacterium.s__*Bifidobacterium_dentium* | yes | 473.4299 | 0.0011 |
| X1CMET2.PWY..N10.formyl.tetrahydrofolate.biosynthesis.g__Blautia.s__*Ruminococcus_gnavus* | yes | 126.3377 | 0.0030 |
| ARGSYN.PWY..L.arginine.biosynthesis.I..via.L.ornithine..g__Blautia.s__*Ruminococcus_gnavus* | yes | 155.6105 | 0.0030 |
| ARGSYNBSUB.PWY..L.arginine.biosynthesis.II..acetyl.cycle..g__Blautia.s__*Ruminococcus_gnavus* | yes | 184.2446 | 0.0023 |
| BRANCHED.CHAIN.AA.SYN.PWY..superpathway.of.branched.amino.acid.biosynthesis.g__Blautia.s__*Ruminococcus_gnavus* | yes | 154.5044 | 0.0031 |
| COA.PWY.1..coenzyme.A.biosynthesis.II..mammalian..g__Blautia.s__*Ruminococcus_gnavus* | yes | 152.4818 | 0.0024 |
| COA.PWY..coenzyme.A.biosynthesis.I.g__Blautia.s__*Ruminococcus_gnavus* | yes | 150.0988 | 0.0026 |
| DTDPRHAMSYN.PWY..dTDP.L.rhamnose.biosynthesis.I.g__Blautia.s__*Ruminococcus_gnavus* | yes | 146.8284 | 0.0015 |
| GLUTORN.PWY..L.ornithine.biosynthesis.g__Blautia.s__*Ruminococcus_gnavus* | yes | 129.8241 | 0.0031 |
| PEPTIDOGLYCANSYN.PWY..peptidoglycan.biosynthesis.I..meso.diaminopimelate.containing..g__Blautia.s__*Ruminococcus_gnavus* | yes | 156.6033 | 0.0026 |
| PWY.1042..glycolysis.IV..plant.cytosol..g__Blautia.s__*Ruminococcus_gnavus* | yes | 170.2212 | 0.0024 |
| PWY.2942..L.lysine.biosynthesis.III.g__Blautia.s__*Ruminococcus_gnavus* | yes | 168.046 | 0.0026 |
| PWY.3841..folate.transformations.II.g__Blautia.s__*Ruminococcus_gnavus* | yes | 135.7699 | 0.0025 |
| PWY.4242..pantothenate.and.coenzyme.A.biosynthesis.III.g__Blautia.s__*Ruminococcus_gnavus* | yes | 148.9855 | 0.0021 |
| PWY.5097..L.lysine.biosynthesis.VI.g__Blautia.s__*Ruminococcus_gnavus* | yes | 158.8982 | 0.0028 |
| PWY.5103..L.isoleucine.biosynthesis.III.g__Blautia.s__*Ruminococcus_gnavus* | yes | 153.8428 | 0.0029 |
| PWY.5667..CDP.diacylglycerol.biosynthesis.I.g__Blautia.s__*Ruminococcus_gnavus* | yes | 137.6698 | 0.0031 |
| PWY.5686..UMP.biosynthesis.g__Blautia.s__*Ruminococcus_gnavus* | yes | 153.4085 | 0.0027 |
| PWY.6121..5.aminoimidazole.ribonucleotide.biosynthesis.I.g__Blautia.s__*Ruminococcus_gnavus* | yes | 150.544 | 0.0028 |
| PWY.6122..5.aminoimidazole.ribonucleotide.biosynthesis.II.g__Blautia.s__*Ruminococcus_gnavus* | yes | 146.362 | 0.0029 |
| PWY.6126..superpathway.of.adenosine.nucleotides.de.novo.biosynthesis.II.g__Blautia.s__*Ruminococcus_gnavus* | yes | 191.424 | 0.0024 |
| PWY.6151..S.adenosyl.L.methionine.cycle.I.g__Blautia.s__*Ruminococcus_gnavus* | yes | 148.139 | 0.0031 |
| PWY.6168..flavin.biosynthesis.III..fungi..g__Faecalibacterium.s__*Faecalibacterium_prausnitzii* | yes | 165.249 | 0.0026 |
| PWY.621..sucrose.degradation.III..sucrose.invertase..g__Bifidobacterium.s__*Bifidobacterium_dentium* | yes | 577.1506 | 0.0016 |
| PWY.6277..superpathway.of.5.aminoimidazole.ribonucleotide.biosynthesis.g__Blautia.s__*Ruminococcus_gnavus* | yes | 146.3629 | 0.0029 |
| PWY.6385..peptidoglycan.biosynthesis.III..mycobacteria..g__Blautia.s__*Ruminococcus_gnavus* | yes | 146.0853 | 0.0030 |
| PWY.6386..UDP.N.acetylmuramoyl.pentapeptide.biosynthesis.II..lysine.containing..g__Blautia.s__*Ruminococcus_gnavus* | yes | 159.5831 | 0.0028 |
| PWY.6387..UDP.N.acetylmuramoyl.pentapeptide.biosynthesis.I..meso.diaminopimelate.containing..g__Blautia.s__*Ruminococcus_gnavus* | yes | 159.868 | 0.0025 |
| PWY.6527..stachyose.degradation.g__Blautia.s__*Ruminococcus_gnavus* | yes | 173.832 | 0.0029 |
| PWY.6609..adenine.and.adenosine.salvage.III.g__Blautia.s__*Ruminococcus_gnavus* | yes | 171.398 | 0.0016 |
| PWY.6700..queuosine.biosynthesis.g__Blautia.s__*Ruminococcus_gnavus* | yes | 110.115 | 0.0026 |
| PWY.6737..starch.degradation.V.g__Blautia.s__*Ruminococcus_gnavus* | yes | 167.253 | 0.0018 |
| PWY.6936..seleno.amino.acid.biosynthesis.g__Blautia.s__*Ruminococcus_gnavus* | yes | 120.057 | 0.0025 |
| PWY.7199..pyrimidine.deoxyribonucleosides.salvage.g__Blautia.s__*Ruminococcus_gnavus* | yes | 133.736 | 0.0026 |
| PWY.7219..adenosine.ribonucleotides.de.novo.biosynthesis.g__Blautia.s__*Ruminococcus_gnavus* | yes | 182.6095 | 0.0026 |
| PWY.7220..adenosine.deoxyribonucleotides.de.novo.biosynthesis.II.g__Blautia.s__*Ruminococcus_gnavus* | yes | 277.0192 | 0.0027 |
| PWY.7221..guanosine.ribonucleotides.de.novo.biosynthesis.g__Blautia.s__*Ruminococcus_gnavus* | yes | 141.313 | 0.0024 |
| PWY.7222..guanosine.deoxyribonucleotides.de.novo.biosynthesis.II.g__Blautia.s__*Ruminococcus_gnavus* | yes | 277.019 | 0.0027 |
| PWY.7229..superpathway.of.adenosine.nucleotides.de.novo.biosynthesis.I.g__Blautia.s__*Ruminococcus_gnavus* | yes | 204.081 | 0.0026 |
| PWY.7357..thiamin.formation.from.pyrithiamine.and.oxythiamine..yeast..g__Blautia.s__*Ruminococcus_gnavus* | yes | 151.121 | 0.0026 |
| PWY.7400..L.arginine.biosynthesis.IV..archaebacteria..g__Blautia.s__*Ruminococcus_gnavus* | yes | 154.296 | 0.0030 |
| PWY0.1296..purine.ribonucleosides.degradation.g__Blautia.s__*Ruminococcus_gnavus* | yes | 161.093 | 0.0014 |
| PWY0.1319..CDP.diacylglycerol.biosynthesis.II.g__Blautia.s__*Ruminococcus_gnavus* | yes | 137.669 | 0.0031 |
| RHAMCAT.PWY..L.rhamnose.degradation.I.g__Blautia.s__*Ruminococcus_gnavus* | yes | 149.167 | 0.0026 |
| RIBOSYN2.PWY..flavin.biosynthesis.I..bacteria.and.plants..g__Faecalibacterium.s__*Faecalibacterium_prausnitzii* | yes | 167.321 | 0.0025 |
| THISYNARA.PWY..superpathway.of.thiamin.diphosphate.biosynthesis.III..eukaryotes..g__Blautia.s__*Ruminococcus_gnavus* | yes | 144.268 | 0.0027 |
| TRPSYN.PWY..L.tryptophan.biosynthesis.g__Blautia.s__*Ruminococcus_gnavus* | yes | 173.449 | 0.0018 |
| ILEUSYN.PWY..L.isoleucine.biosynthesis.I..from.threonine..g__Blautia.s__*Ruminococcus_gnavus* | yes | 163.440 | 0.0034 |
| PWY.7111..pyruvate.fermentation.to.isobutanol..engineered..g__Blautia.s__*Ruminococcus_gnavus* | yes | 199.953 | 0.0034 |
| PYRIDNUCSYN.PWY..NAD.biosynthesis.I..from.aspartate..g__Blautia.s__*Ruminococcus_gnavus* | yes | 129.144 | 0.00352 |
| PWY4FS.7..phosphatidylglycerol.biosynthesis.I..plastidic..g__Blautia.s__*Ruminococcus_gnavus* | yes | 119.961 | 0.00364 |
| PWY4FS.8..phosphatidylglycerol.biosynthesis.II..non.plastidic..g__Blautia.s__*Ruminococcus_gnavus* | yes | 119.961 | 0.00364 |
| PHOSLIPSYN.PWY..superpathway.of.phospholipid.biosynthesis.I..bacteria..g__Blautia.s__*Ruminococcus_gnavus* | yes | 128.775 | 0.00371 |
| VALSYN.PWY..L.valine.biosynthesis.g__Blautia.s__*Ruminococcus_gnavus* | yes | 160.932 | 0.00398 |
| PWY.7220..adenosine.deoxyribonucleotides.de.novo.biosynthesis.II.g__Faecalibacterium.s__*Faecalibacterium_prausnitzii* | yes | 120.3413 | 0.00441 |
| PWY.7222..guanosine.deoxyribonucleotides.de.novo.biosynthesis.II.g__Faecalibacterium.s__*Faecalibacterium_prausnitzii* | yes | 120.3413 | 0.00441 |
| PWY.7242..D.fructuronate.degradation.g__Faecalibacterium.s__*Faecalibacterium_prausnitzii* | yes | 120.7312 | 0.00481 |
| GALACTUROCAT.PWY..D.galacturonate.degradation.I.g__Faecalibacterium.s__*Faecalibacterium_prausnitzii* | yes | 129.3251 | 0.00544 |
| PWY.6507..4.deoxy.L.threo.hex.4.enopyranuronate.degradation.g__Faecalibacterium.s__*Faecalibacterium_prausnitzii* | yes | 114.3616 | 0.00554 |
| PWY.6897..thiamin.salvage.II.g__Faecalibacterium.s__*Faecalibacterium_prausnitzii* | yes | 100.8492 | 0.00608 |
| PWY.5177..glutaryl.CoA.degradation.g__Faecalibacterium.s__*Faecalibacterium_prausnitzii* | yes | 143.8907 | 0.00643 |
| PWY.5695..urate.biosynthesis.inosine.5..phosphate.degradation.g__Faecalibacterium.s__*Faecalibacterium_prausnitzii* | yes | 102.5299 | 0.00711 |
| PWY.6737..starch.degradation.V.g__Faecalibacterium.s__*Faecalibacterium_prausnitzii* | yes | 183.4145 | 0.00748 |
